# Lifetime Variations in Prolactin Expression in the Hippocampus and Dentate Gyrus of the Rat

**DOI:** 10.3390/ijms26157299

**Published:** 2025-07-28

**Authors:** Marta Carretero-Hernández, Elisa Herráez, Leonardo Catalano-Iniesta, David Hernández-González, David Díez-Castro, Ana E. Rodríguez-Vicente, Josefa García-Barrado, Teresa Vicente-García, Miguel Robles-García, Enrique J. Blanco, José Carretero

**Affiliations:** 1 Department of Human Anatomy and Histology, Faculty of Medicine, University of Salamanca, 37007 Salamanca, Spain; leonardo.catalano@usal.es (L.C.-I.); davidhnz@usal.es (D.H.-G.); dadicas@usal.es (D.D.-C.); anita82@usal.es (A.E.R.-V.); tvicente@usal.es (T.V.-G.); mroblesgarcia@usal.es (M.R.-G.); ejbb@usal.es (E.J.B.); jcar@usal.es (J.C.); 2 Center of Experimental Orthopaedics, Saarland University, Kirrberger Str. Building 37, 66424 Homburg, Germany; 3 Laboratory of Neuroendocrinology, Institute of Neuroscience of Castilla y León, University of Salamanca, 37007 Salamanca, Spain; barrado@usal.es; 4 Laboratory of Neuroendocrinology and Obesity, Biosanitary Institute of Salamanca (IBSAL), 37007 Salamanca, Spain; 5 Department of Physiology and Pharmacology, Faculty of Medicine, University of Salamanca, 37007 Salamanca, Spain; elisah@usal.es; 6 Experimental Hepatology and Drug Targeting (HEVEPHARM) Group, Institute of Biomedical Research of Salamanca (IBSAL), University of Salamanca, 37007 Salamanca, Spain; 7 National Institute for the Study of Liver and Gastrointestinal Diseases (CIBERehd, “Instituto de Salud Carlos III”), 28029 Madrid, Spain

**Keywords:** hippocampus (Ammon’s horn), dentate gyrus, prolactin, sex, aging

## Abstract

Prolactin is a hormone with demonstrated roles in the brain, including neurogenesis, neuroprotection, learning, stress response or memory consolidation. To determine the prolactin expression in the rat hippocampus during aging and to resolve some controversies related to the presence of prolactin in the hippocampus, the aim of this study was to analyze whether changes occur in the expression of prolactin during different stages of life. To determine this, we designed an experimental study in which we analyzed the expression and location of prolactin in the rat hippocampus, Ammon’s horn and Dentate Gyrus, during different stages of life (prepubertal, postpubertal, young adult, adult and old) and checked if there are differences related to sex. Overall, the results obtained show that prolactin is present in the rat hippocampus and that prolactin is synthesized, as deduced from the findings obtained via ELISA, immunohistochemistry, qPCR and in situ hybridization. After analyzing the correlation between serum and hippocampal prolactin levels and comparing the amounts of *Prl* mRNA and the hormone, the results obtained suggest that hippocampal prolactin has a dual origin: local synthesis of the hormone and its passage from the blood. On the other hand, the amounts of prolactin and its mRNA in the hippocampus vary with sex and age, suggesting the existence of age-related sexual dimorphism. The results obtained suggest that hippocampal aging is related to a decrease in the hippocampal prolactin system, which helps to better understand brain aging.

## 1. Introduction

Prolactin is a hormone mainly synthesized in the pituitary gland and released into the bloodstream through the pituitary portal system. It acts on specific receptors and has been implicated in more than 300 biological actions. While its roles in lactation and reproduction are widely known, it also has important effects at the brain level, including the stimulation of neurogenesis, modulation of stress and anxiety, calcium transport and regulation of the immune system [1,2,3].

In conditions where prolactin levels rise physiologically, such as during pregnancy and lactation or following exogenous hormone administration, the hormone appears to exert neuroprotective effects. Prolactin has been shown to prevent neuronal injury or death caused by stress, ischemic states or exposure to neurotoxins [4,5,6,7], mainly acting on pyramidal neurons in Ammon’s horn.

The demonstration of the modulation of Tau phosphorylation during pregnancy and lactation [8] or the decrease in b-amyloid aggregation and Tau phosphorylation in the hippocampus, described in transgenic animal models of amyloidosis after administration of prolactin analogs [9], also supports the action of prolactin as a neuroprotector.

Due to its molecular size and protein structure, prolactin is unable to cross the blood–brain barrier. However, since the choroid plexuses are rich in prolactin receptors, several studies have hypothesized that circulating prolactin may enter the cerebrospinal fluid (CSF) through a receptor-mediated transport mechanism involving the prolactin receptor [1,2,3,6,10,11].

Nevertheless, the finding that prolactin receptor knockout animals exhibit CSF prolactin levels comparable to those of control animals [12] seriously questioned this hypothesis. This observation raised the possibility of a specific vascular carrier independent of CSF involvement.

The observed correlation between brain and circulating prolactin levels [6,13,14] suggests that prolactin in the brain may originate from the bloodstream. Nonetheless, studies in hypophysectomized animals have shown that prolactin remains detectable in the brain [15], pointing to the existence of a brain origin.

The translocation of prolactin from the bloodstream into the brain cannot be entirely ruled out, and several studies have shown the brain synthesis of the hormone [15,16,17,18,19], which raises the possibility that prolactin plays an auto-paracrine role in the brain.

Although scientific literature reports conflicting data regarding age-related variations in circulating prolactin levels [20,21,22], there is a lack of studies examining whether prolactin expression in the hippocampus varies across different life stages. It remains unclear if hippocampal prolactin correlates with blood prolactin levels, and whether local prolactin synthesis occurs in the hippocampus or undergoes changes.

Thus, to contribute to the understanding of prolactin dynamics in the rat hippocampus during aging and to clarify existing controversies regarding the presence of prolactin in this brain region, this study aimed to investigate potential changes in prolactin expression across different life stages in rats. For this purpose, an experimental analysis was carried out to assess the expression and distribution of prolactin in the rat hippocampus—specifically in Ammon’s horn and the Dentate Gyrus—across different age groups, while also considering potential sex-related differences.

## 2. Results

### 2.1. Hippocampal Prl mRNA Detection via qPCR

**Figure 1:**
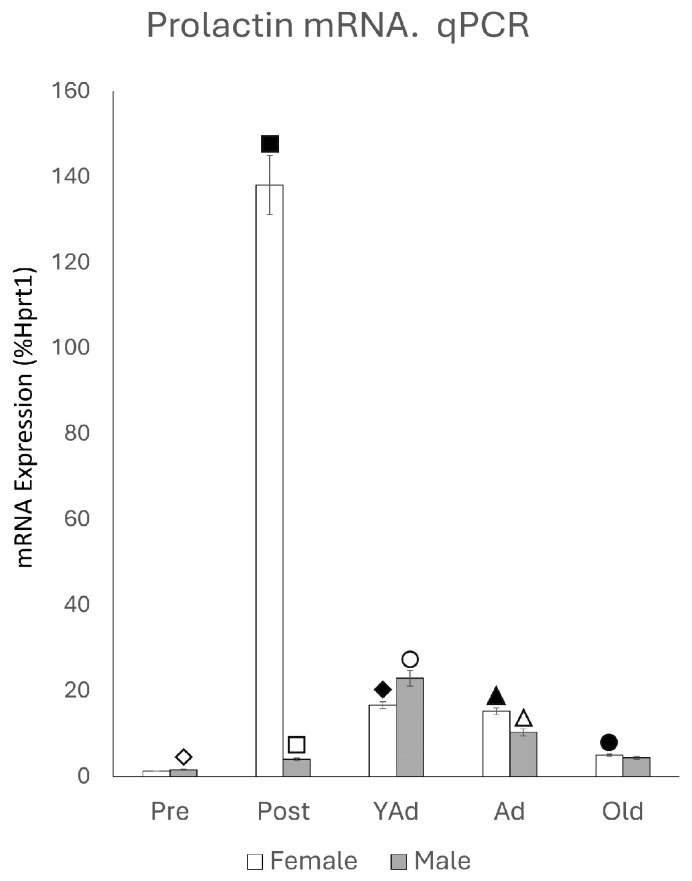
qPCR results for *Prl* mRNA in the hippocampi of female and male rats at different life stages (n: 5 animals per group studied). Two-tailed ANOVA results (Tukey’s test): ◇ *p* < 0.05 in relation to prepubertal females, ■ *p* < 0.001 relative to all other groups studied, ☐ *p* < 0.05 in relation to prepubertal males, ◆ *p* < 0.01 relative to prepubertal females, ○ *p* < 0.01 in relation to postpubertal males and *p* < 0.05 in relation to young adult females, ▲ *p* < 0.01 relative to prepubertal females, △ *p* < 0.01 in relation to old males and *p* < 0.05 in relation to adult females, and ● *p* < 0.01 in relation to adult females and *p* < 0.05 in relation to old males.

*Prl* mRNA was detected in both sexes across all age groups. However, the expression patterns differed between males and females (Figure 1). In females, the most striking finding was a marked increase in *Prl* mRNA levels in the postpubertal group, which exhibited a seven-fold elevation compared to all other female groups (*p* < 0.001). In contrast, prepubertal females showed low *Prl* mRNA expression. Young adult and adult females showed comparable levels, both significantly higher than those observed in the prepubertal and old groups (*p* < 0.01).

In males, *Prl* mRNA levels increased from the prepubertal stage to the young adult stage, followed by a decline with advancing age. Prepubertal males exhibited the lowest expression levels, although these were significantly higher than those observed in age-matched females (*p* < 0.05). The levels of *Prl* mRNA in postpubertal males were higher than in prepubertal males (*p* < 0.05), but their levels remained markedly lower than those of postpubertal females (*p* < 0.001). The highest values of *Prl* mRNA in males were found in young adult animals that showed a significant increase (*p* < 0.01) with respect to postpubertal animals and exceeded those in young adult females (*p* < 0.05). In adult males, a significant decline was observed compared to the young adult group (*p* < 0.01), and *Prl* mRNA levels were lower than those in females of the same age (*p* < 0.05). A further significant reduction occurred in old males compared to adult males (*p*< 0.01), and expression levels were also significantly lower than those found in old females (*p* < 0.05). In summary, the highest values were found in postpubertal female rats and young adult male rats. Except at the prepubertal and young adult stages, female rats exhibited higher *Prl* mRNA levels than males across the remaining life stages.

Interestingly, the hippocampal samples used to optimize the qPCR and in situ hybridization protocols—obtained from lactating young adult female rats that were nursing pups—showed *Prl* mRNA levels 77 times higher than those of the housekeeping gene *Hprt1*. Compared to age-matched females in our experimental groups, *Prl* mRNA levels in these lactating rats were more than 464 times higher (*p* < 0.001).

### 2.2. Hippocampal Prl mRNA Detection via In Situ Hybridization, Densitometric Analysis

**Figure 2:**
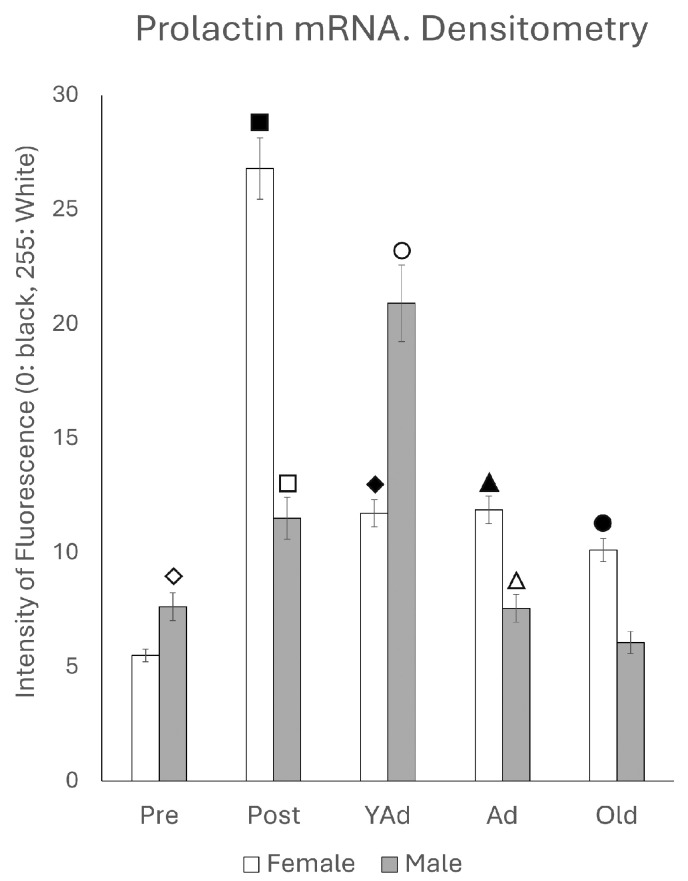
Densitometric values of the intensity of fluorescence for *Prl* mRNA (black = 0 white = 255) found in the total of hippocampal structures studied (n: 5 animals per group studied). (Pre: prepubertal animals, Post: postpubertal animals, YAd: young adult animals, Ad: adult animals, and Old: old animals). Two-tailed ANOVA results (Tukey’s test): ◇ *p* < 0.05 in relation to prepubertal females, *p* < 0.05 in relation to postpubertal and old males, *p* < 0.01 in relation to young adult males, ■ *p* < 0.001 relative to all other groups studied, ☐ *p* < 0.05 in relation to prepubertal and adult males, *p* < 0.01 in relation to young adult and old males, ◆ *p* < 0.01 relative to prepubertal females and young adult males, ○ *p* < 0.01 in relation to other groups of males and in relation to young adult females, ▲ *p* < 0.01 in relation to adult males, △ *p* < 0.05 in relation to old males, and ● *p* < 0.01 in relation to old males.

Densitometric analysis of the entire dorsal hippocampus, including horn of Ammon and dentate gyrus, showed mean gray values for each sex and age stage that followed a pattern consistent with the results obtained via qPCR (Figure 2).

Accordingly, the highest mean gray values were observed in postpubertal females and young adult males. Except for the prepubertal and young adult stages, mean gray values were significantly higher in females than in males across the remaining age groups. Statistical significance for each of the analyzed regions is detailed below.

### 2.3. Prolactin-Synthesizing Cells: Regional Analysis of In Situ Hybridization

#### 2.3.1. CA1 Region

**Figure 3:**
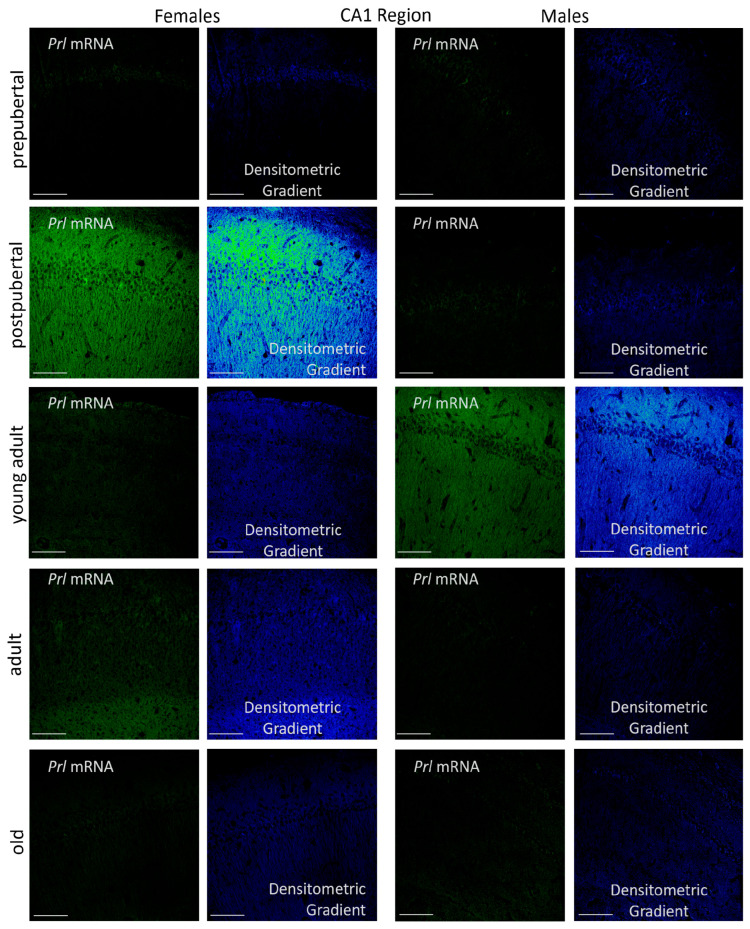
Confocal microphotographs of in situ hybridization for *Prl* mRNA in the CA1 region of females and males at different stages of life. Sexual dimorphic expression was evident, mainly in postpubertal females and young adult males. The expression, with differences throughout life, was found in all CA1 layers, with greater intensity in the pyramidal layer. The images of the densitometric gradients highlight the reaction intensity in each of the layers in the different groups of animals studied. Scale bar: 116 µm.

*Prl* mRNA was clearly detected in the oriens, pyramidal, and radiatum layers of the hippocampal CA1 region (Figure 3). In prepubertal females, the majority of *Prl* mRNA-positive neurons were in the pyramidal layer of this region, exhibiting a punctiform or granular reaction in their cytoplasm. Strikingly, among these homogeneous and weakly reactive pyramidal neurons, a subset of more reactive and isolated cells appeared indistinctly close to the radiated or molecular layer. Additionally, isolated and scattered secondary neurons were observed in the molecular layer of the CA1 region of these animals, with smaller-sized neurons detected in the radiatum layer. The dendrites and axons of these neurons hardly reacted; however, densitometric analysis showed a gradient of higher reaction intensity in areas closer to the pyramidal layer within the oriens and radiatum layers.

Very different results were observed in postpubertal females. Practically, all pyramidal neurons in the CA1 region were positive for *Prl* mRNA, and the positivity in dendrites and axons was very striking, affecting the oriens and radiatum layers. According to densitometric analysis, fluorescence intensity in these structures was significantly higher than in prepubertal females. Additionally, a subset of secondary neurons within the oriens, radiatum and even the lacunous layers were strongly positive in these animals. The hyperexpression of *Prl* mRNA observed in postpubertal females decreased in young adult females and declined further in adult and older females. In all cases, a punctiform reaction appeared, complicating the clear identification and delimitation of individual neurons. These animals had hardly any positive secondary neurons, with *Prl* mRNA expression largely restricted to pyramidal neurons. Densitometric gradients showed a reduction in *Prl* mRNA expression from young to old adulthood.

The appearance and distribution pattern of *Prl* mRNA in the CA1 region of prepubertal males resembled that observed in female rats. The signal was predominantly restricted to the pyramidal layer, where a subset of highly reactive neurons appeared, exhibiting positive extensions toward both the radiatum and oriens layers. However, the reaction was not homogeneous, as some exhibited greater reactivity than others. In contrast to the pattern observed in females, the morphological distribution in postpubertal males resembled that of prepubertal males, with a predominant reaction in the pyramidal layer, where highly positive neurons appeared. However, postpubertal males showed fewer reactive prolongations and a limited number of positive secondary neurons, mainly located in the radiatum layer.

Moreover, unlike females, young adult males exhibited the highest *Prl* mRNA expression. The increased reaction was distributed uniformly across all CA1 layers, with many strongly positive neurons in the pyramidal layer and widespread positivity in neuronal prolongations within both the radiatum and oriens layers. No secondary neurons were found to be positive in these animals.

**Figure 4:**
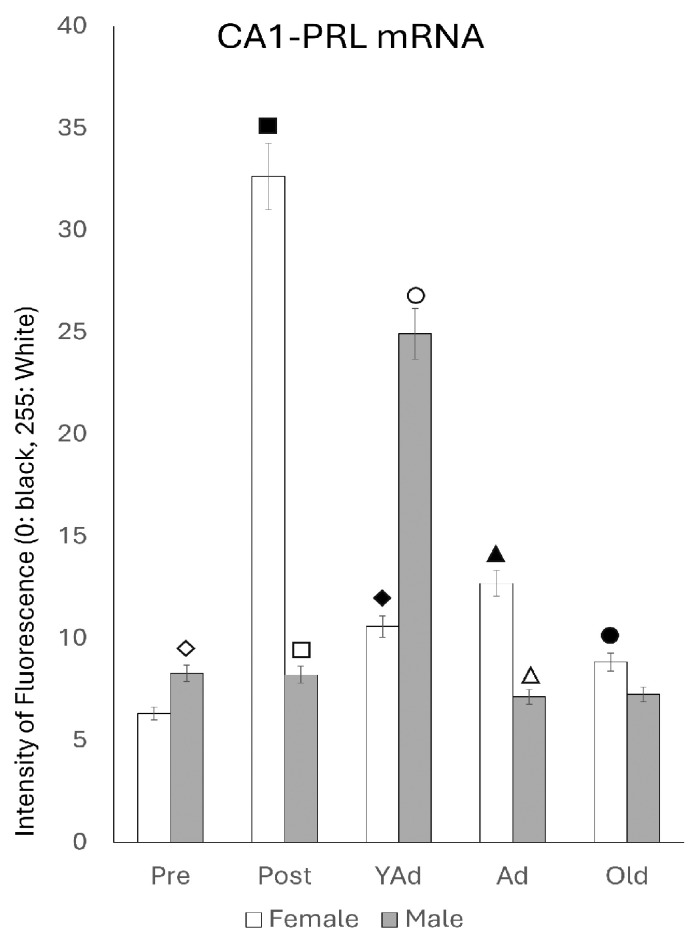
Densitometric values of the intensity of fluorescence for *Prl* mRNA (black = 0 and white = 255) found in the CA1 region (n: 5 animals per group studied). (Pre: prepubertal animals, Post: postpubertal animals, YAd: young adult animals, Ad: adult animals, and Old: old animals). Two-tailed ANOVA results (Tukey’s test): ◇ *p* < 0.05 in relation to prepubertal females, *p* < 0.05 in relation to postpubertal and old males, *p* < 0.01 in relation to young adult males, ■ *p* < 0.001 relative to all other groups studied, ☐ *p* < 0.05 in relation to prepubertal and adult males, *p* < 0.01 in relation to young adult and old males, ◆ *p* < 0.01 relative to prepubertal females and young adult males, ○ *p* < 0.01 in relation to other groups of males and in relation to young adult females, ▲ *p* < 0.01 in relation to adult males, △ *p* < 0.05 in relation to old males, and ● *p* < 0.01 in relation to old males.

Densitometric analysis of fluorescence intensity in the three layers of the CA1 region revealed sexually dimorphic patterns with variations related to the age of the animals. These differences followed a similar trend across all layers analyzed (Figure 4). In female rats, the intensity was low at the prepubertal stage; it increased significantly in the postpubertal animals (*p* < 0.001), decreased again in young adults (*p* < 0.001), showed a slight increase in adults (*p* < 0.05) and declined in old females (*p* < 0.01). In males, prepubertal animals showed higher intensity values than females of the same age (*p* < 0.05). However, postpubertal males exhibited significantly lower intensity than females of the same group (*p* < 0.001), with values like those observed in prepubertal males. Among all male groups, young adults had the highest intensity of reaction (*p* < 0.01). The intensity of reaction decreased in adult and old males, reaching lower values, though not significantly different from those observed in pre- and postpubertal males. Except in prepubertal males (*p* < 0.05) and young adults (*p* < 0.01), fluorescence intensity was higher in females than in males at all other stages—specifically in postpubertal (*p* < 0.001), adult (*p* < 0.01) and old animals (*p* < 0.05).

#### 2.3.2. CA3 Region

**Figure 5:**
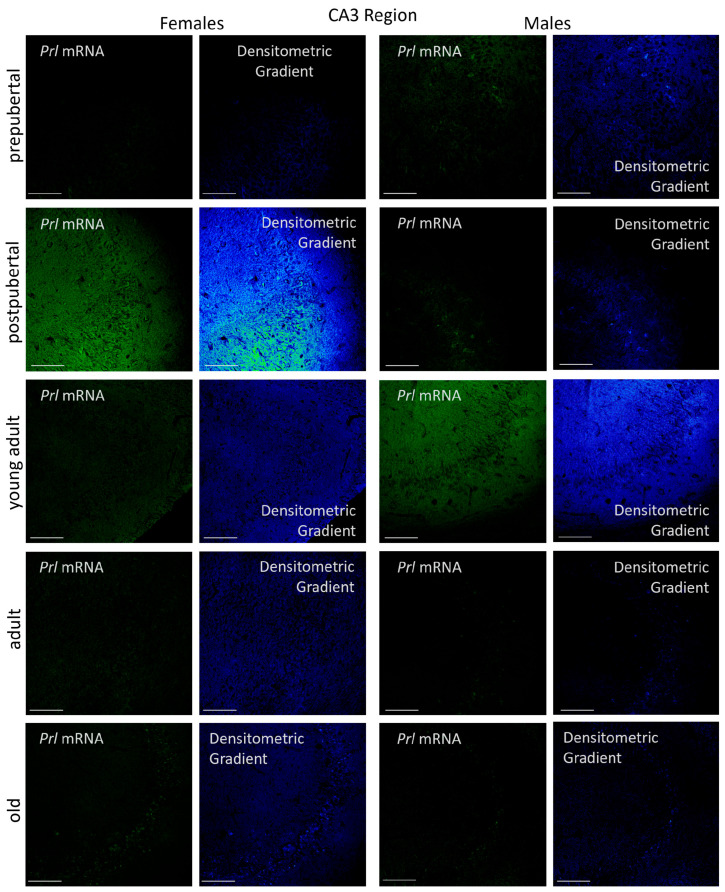
Confocal microphotographs of in situ hybridization for *Prl* mRNA in the CA3 region of females and males at the different stages of life. Like to those observed in the CA1 region, the sexual dimorphic expression was evident, mainly in postpubertal females and young adult males. The expression, with differences a long of the life, was found mainly in the pyramidal layer of the CA3 region. The images of the densitometric gradients highlight the reaction intensity in each of the layers in the different groups of animals studied. Scale bar: 116 µm.

All pyramidal neurons in the CA3 region of postpubertal female rats were positive for *Prl* mRNA, showing a highly reactive cytoplasm. This reaction was also observed in some neurons located in the oriens and radiate layers (Figure 5). The *Prl* mRNA expression extended into the dendrites and axons of these layers, resulting in a homogeneous and highly intense signal, as confirmed by densitometric gradient analysis. The densitometric gradient revealed that the prepubertal females had the lowest levels of reactivity. Although a few isolated neurons in the pyramidal layer of these animals appeared reactive, the overall reaction was limited to a punctiform pattern in the pyramidal layer and in some areas of the radiated layer. In young adult and adult females, a punctiform reaction was also observed across all three layers studied, although they were more concentrated in the neuronal somas of the pyramidal layer. In old females, reactive neuronal somas were still present in the pyramidal layer, but the reaction in the dendrites or axons of the oriens and radiated layers was almost negligible.

The presence of *Prl* mRNA in the hippocampal CA3 region was evident across the three layers analyzed: oriens, pyramidal, and radiate. In all male age groups studied, the majority of *Prl* mRNA-positive neurons were in the pyramidal layer and exhibited a punctiform or granular reaction in the cytoplasm. In prepubertal, postpubertal, and young adult males, secondary neurons were observed as isolated cells scattered in the molecular layer, and smaller neurons were occasionally detected in the radiated layer. In old males, The CA3 region contained very few positive neurons, although a punctiform reaction appeared in the pyramidal layer. Overall, dendrites and axons of these neurons hardly reacted, and the densitometric gradient analysis indicated that the areas adjacent to the pyramidal layer—specifically within the oriens and radiated layers—had a greater reaction intensity. The highest density of reactive axons and dendrites in CA3 was found in young adult males, followed by prepubertal males, and was almost completely absent in adult and old males.

**Figure 6:**
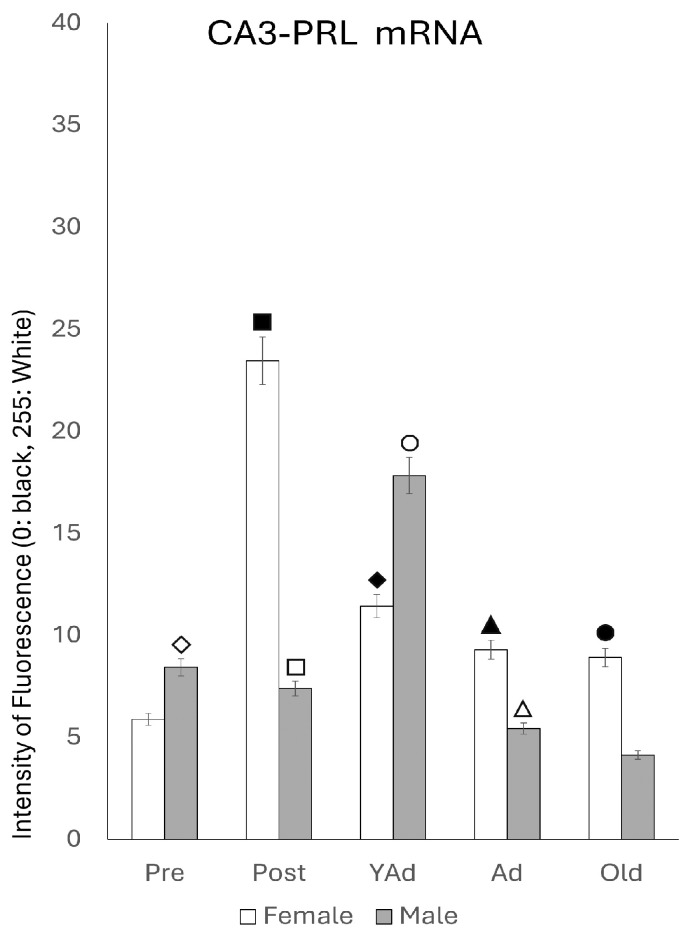
Densitometric values of the intensity of fluorescence for *Prl* mRNA (black = 0 and white = 255) found in the CA3 region (n: 5 animals per group studied). (Pre: prepubertal animals, Post: postpubertal animals, YAd: young adult animals, Ad: adult animals, and Old: old animals). Two-tailed ANOVA results (Tukey’s test): ◇ *p* < 0.05 in relation to prepubertal females, ■ *p* < 0.001 in relation to the other groups of females studied and postpubertal males, ☐ *p* < 0.001 in relation to postpubertal females, ◆ *p* < 0.01 in relation to prepubertal females and *p* < 0.05 in relation to adult and old females, ○ *p* < 0.01 in relation to pre- and postpubertal males, *p* < 0.005 in relation to adult and old males and *p* < 0.05 in relation to young adult females, ▲ *p* < 0.05 in relation to prepubertal females and adult males, △ *p* < 0.01 in relation to pre- and postpubertal males, and ● *p* < 0.05 in relation to prepubertal females and old males.

Densitometric analysis of *Prl* mRNA expression in the CA3 region showed that, when considering the entire area, the reaction intensity was generally lower than that observed in the CA1 region across most stages of life. However, the pattern of variations in reaction intensity (Figure 6) resembled that described for CA1. In females, the lowest intensity values were observed in prepubertal females, while the highest were shown in postpubertal females (*p* < 0.001 vs. all other female groups). Young adult females had significantly lower values than postpubertal females (*p* < 0.001) but higher than those found in prepubertal (*p* < 0.01), adult and old females (*p* < 0.05). In adult and old females, no significant differences were observed between adult and old females, although both groups displayed higher intensity of fluorescence than prepubertal females (*p* < 0.05). In males, prepubertal animals showed significantly higher reaction intensity compared to females of the same age (*p* < 0.05). In contrast, postpubertal males presented markedly lower intensity values than postpubertal females (*p* < 0.001), with no significant differences with prepubertal males. Among groups, young adults showed the highest fluorescence intensity (*p* < 0.01 vs. pre- and postpubertal males; *p* < 0.005 vs. adult and old males). No significant differences were observed between adult and old males, although both groups had significantly lower values than the ones found in pre- and postpubertal males (*p* < 0.01). As in the CA1 region, significant differences in *Prl* mRNA expression between females and males appeared at all stages of life. Except for prepubertal and young adult males, the intensity of reaction was higher in females than in males across all other stages: (*p* < 0.001 for postpubertal animals, *p* < 0.01 for prepubertal, young adults, adults and old animals).

#### 2.3.3. Dentate Gyrus 

**Figure 7:**
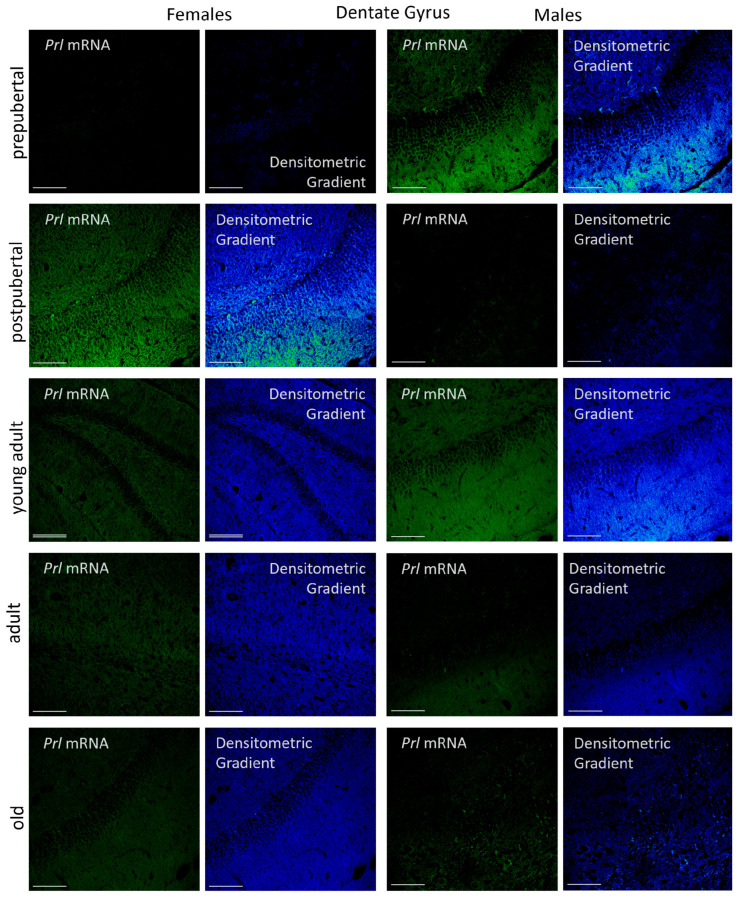
Confocal microphotographs of in situ hybridization for *Prl* mRNA in the DG region of females and males at different stages of life. Sexual dimorphic expression was evident, mainly in prepubertal males, postpubertal females and young adult males. In prepubertal and postpubertal animals and in young adult males, positive subgranular cells were found. Expression, with differences throughout life, was found mainly in molecular and granular layers, except in adult animals. The images of the densitometric gradients highlight the reaction intensity in each of the layers across the different groups of animals studied. Scale bar: 116 µm, Double Scale bar: 200 µm.

In the dentate gyrus, three different areas were analyzed: the dorsal, the ventral and the beak. Apart from the morphological differences, no significant differences in *Prl* mRNA signal intensity were observed in any of the groups analyzed (Figure 7).

In females, the greatest reactivity—affecting the neuronal somas in the granular layer and fibers in both the molecular and polymorphic layers—was observed in postpubertal animals. In this group, subgranular cells arranged between the granular and polymorphic layers exhibited stronger signal intensity than the rest of the cells in the dentate gyrus. Prepubertal females had a reaction in the granular layer and in secondary neurons of the polymorphic layer, with some positive subgranular cells; however, the overall reaction intensity was lower than that seen in postpubertal females. The oriens and polymorphic layers had hardly any reactive fibers. In young adults, the reaction affected all three layers, although the granular layer was less reactive than that at earlier stages, and the number of positive subgranular cells was reduced. Within the molecular layer, two distinct zones were identified: a more reactive marginal zone and an inner zone adjacent to the granular layer. In adult and old females, reactivity in the molecular and polymorphic layers progressively decreased with age, as did the presence of subgranular cells. Old females showed alternating positive and negative zones in the granular layer, and some secondary neurons in the polymorphic layer were strongly positive. All these differences became evident when analyzing the densitometric gradients.

In male rats, the layered reaction patterns of *Prl* mRNA hybridization between pre- and postpubertal stages appeared inverted compared to females. The highest intensities were observed in prepubertal and young adult males. In prepubertal males, almost all granular cells were positive, and the fibers in the molecular layer were very positive. Intensely reactive secondary neurons were also present in the polymorphic layer, and highly positive subgranular cells stood out, some of which extended into the granular layer. In postpubertal males, positivity in the fibers of the molecular and polymorphic layers decreased markedly. Fewer reactive granular cells were observed, and subgranular positive cells were absent, although isolated positive secondary neurons remained in the polymorphic layer. In young adults, the hybridization signal increased again, showing strong positivity across all three layers, even higher than that in prepubertal animals, but without subgranular cells or positive secondary neurons. As in females, hybridization signal intensity in the three layers of the dentate gyrus decreased with age in males. The pattern of reaction in adult males resembled that of prepubertal males, but with lower reaction intensity and no subgranular cells. In old males, the signal appeared in scattered granular cells—some strongly positive along with others unreactive (see densitometric gradient). Subgranular positive cells were absent, while there were secondary neurons in the polymorphic layer.

**Figure 8:**
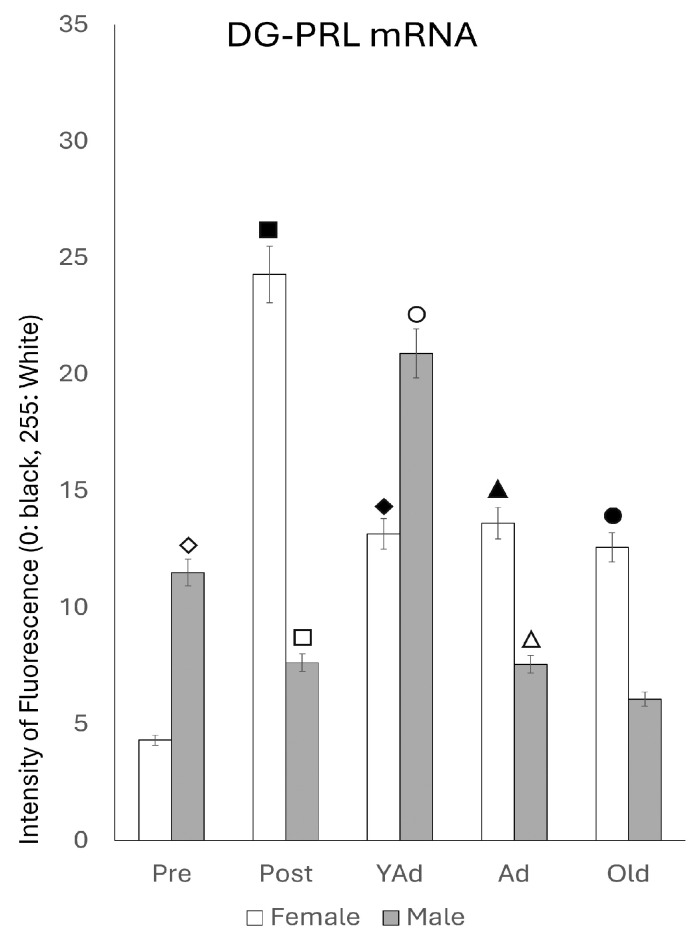
Densitometric values of the intensity of fluorescence for *Prl* mRNA (black = 0 and white = 255) found in the DG region (n: 5 animals per group studied). (Pre: prepubertal animals, Post: postpubertal animals, YAd: young adult animals, Ad: adult animals, and Old: old animals). Two-tailed ANOVA results (Tukey’s test): ◇ *p* < 0.005 in relation to prepubertal females, ■ *p* < 0.001 in relation to prepubertal females and *p* < 0.01 in relation to young adult, adult and old females, ☐ *p* < 0.001 in relation to postpubertal females and *p* < 0.01 in relation to prepubertal males, ◆ *p* < 0.005 in relation to prepubertal females, ○ *p* < 0.005 in relation to postpubertal, adult and old males and *p* < 0.01 in relation to prepubertal females, ▲ *p* < 0.005 in relation to prepubertal females, △ *p* < 0.005 in relation to young adult males, and *p* < 0.01 in relation to prepubertal males and adult females, and ● *p* < 0.05 in relation to prepubertal females and *p* < 0.01 in relation to old males.

The densitometric study of *Prl* mRNA fluorescence intensity in the dentate gyrus revealed age- and sex-dependent differences that, in addition, showed a distinct pattern compared to those observed in the CA1 and CA3 regions (Figure 8).

In female rats, the lowest fluorescence intensity was observed in prepubertal animals, followed by a significant increase in postpubertal females (*p* < 0.001). This intensity decreased in young adult females (*p* < 0.01) and remained stable throughout life in adult and old females. No significant differences were found among young adults, adults and old females; however, the values in these three stages of life were significantly higher (*p* < 0.005) than in the prepubertal stage. In males, prepubertal animals showed significantly higher mean gray values than prepubertal females (*p* < 0.005) and postpubertal males (*p* < 0.01). Postpubertal males exhibited significantly lower values than postpubertal females (*p* < 0.001). A marked increase in the reaction intensity was observed in young adult males (*p* < 0.005). Although the values in these animals were lower than those in prepubertal females (*p* < 0.05), the difference between the two peaks (prepubertal and young adult) was smaller than that observed in CA1 and CA3. The densitometric values decreased significantly (*p* < 0.005) from the young adult to the adult stage, with no further significant changes in old males. Young adult males displayed higher intensity than young adult females (*p* < 0.01). In contrast, adult and old males showed significantly lower values than prepubertal males, as well as adult and old females (*p* < 0.01).

### 2.4. Serum and Hippocampal Prolactin Detection via ELISA

#### 2.4.1. Serum Prolactin Levels

**Figure 9:**
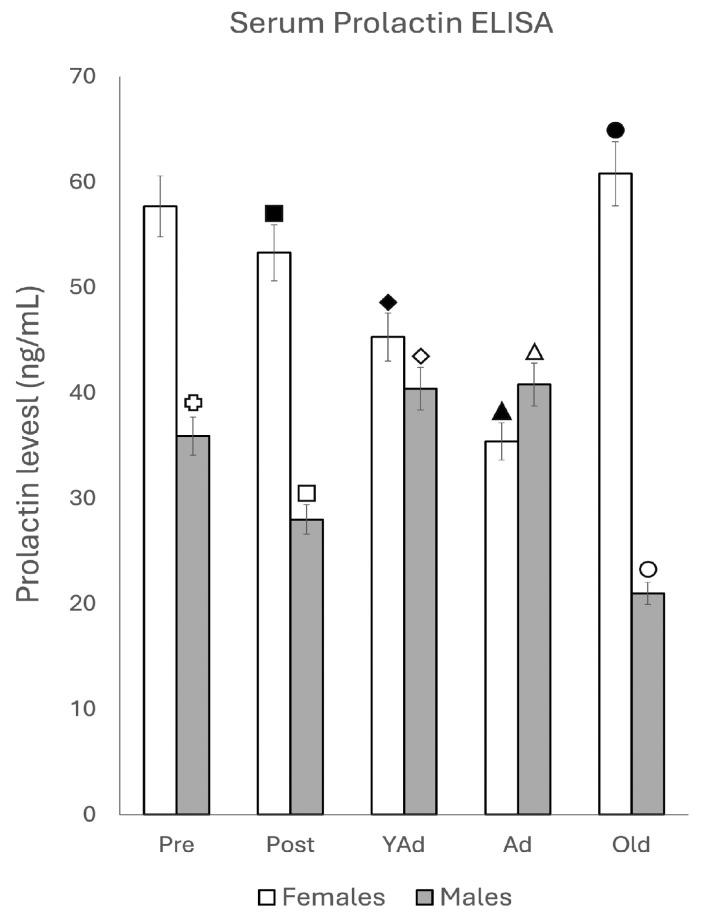
Serum prolactin levels determined via ELISA (n: 5 animals per group studied). (Pre: prepubertal animals, Post: postpubertal animals, YAd: young adult animals, Ad: adult animals, and Old: old animals). Two-tailed ANOVA results (Tukey’s test): 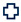 *p* < 0.01 in relation to prepubertal females, ■ *p* < 0.01 relative to postpubertal males, ☐ *p* < 0.05 in relation to prepubertal males, ◆ *p* < 0.01 in relation to prepubertal females and *p* < 0.05 in relation to postpubertal females, ◇ *p* < 0.05 in relation to postpubertal males, ▲ *p* < 0.05 in relation to young adult females, △ *p* < 0.05 in relation to postpubertal males, ● *p* < 0.005 in relation to adult females, *p* < 0.01 in relation to young adult females and *p* < 0.05 in relation to postpubertal females, and ○ *p* < 0.01 in relation to prepubertal, young adult and adult males and *p* < 0.05 in relation to postpubertal males.

Serum prolactin levels in female animals (Figure 9) decreased progressively from prepubertal to adult animals. Differences between prepubertal and postpubertal females were not statistically significant. In young adult females, prolactin levels were significantly lower than in both prepubertal (*p* < 0.01) and postpubertal females (*p* < 0.05). Adult females showed a further reduction compared to young adults (*p* < 0.05). In old females, serum prolactin levels increased significantly compared to adult (*p* < 0.005), young adult (*p* < 0.01) and postpubertal females (*p* < 0.05). In male rats, serum prolactin levels were significantly lower in postpubertal males compared to prepubertal males (*p* < 0.01). No significant variations were observed between young adult and adult males; however, prolactin levels in both stages were significantly higher than in postpubertal males (*p* < 0.05). Serum prolactin levels decreased in old males compared to prepubertal, young adult and adult males (*p* < 0.01), as well as compared to postpubertal males (*p* < 0.05).

#### 2.4.2. Hippocampal Prolactin Levels

**Figure 10:**
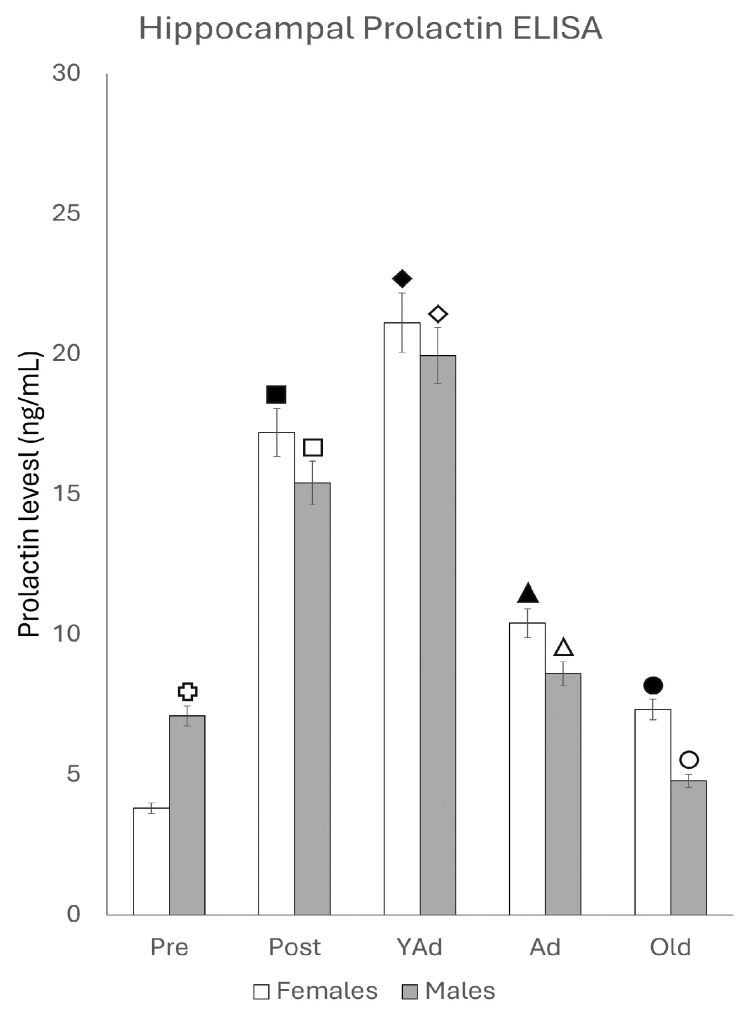
Hippocampal prolactin levels determined via ELISA (n: 5 animals per group studied). (Pre: Prepubertal animals, Post: Postpubertal animals, YAd: Young adult animals, Ad: Adult animals, and Old: Old animals). Two-tailed ANOVA results (Tukey’s test): 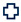 *p* < 0.01 in relation to prepubertal females, ■ *p* < 0.01 relative to prepubertal females, ☐ *p* < 0.01 relative to prepubertal males, ◆ *p* < 0.05 in relation to postpubertal females, ◇ *p* < 0.01 in relation to prepubertal male and *p* < 0.05 in relation to postpubertal males, ▲ *p* < 0.01 in relation to young adult females, △ *p* < 0.01 in relation to young adult males, ● *p* < 0.05 in relation to adult females and *p* < 0.01 in relation to old males, and ○ *p* < 0.01 in relation to adult males old females.

The analysis of the levels of hippocampal prolactin (Figure 10) showed that, except in prepubertal animals, females exhibited higher values that males at all ages, although the differences between females and males in the postpubertal, young adult and adult stages were not statistically significant. In contrast, old females showed significantly higher levels than males (*p* < 0.01). Prepubertal males displayed significantly higher levels of hippocampal prolactin than prepubertal females (*p* < 0.01). When analyzed separately by sex, hormone levels increased gradually in females, reaching a peak in their young adult age and decreased again with age: *p* < 0.05 in relation to postpubertal females, *p* < 0.01 in relation to adult and old females, and *p* < 0.005 in relation to prepubertal females.

A similar pattern was observed in male rats: hippocampal prolactin levels increased from prepubertal to young adult males (*p* < 0.01), decreased significantly in adult males (*p* < 0.01) and reached the lowest levels in old males (*p* < 0.01).

As shown in Table 1, no significant correlation was found between serum and hippocampal prolactin levels in either sex, which was more evident in females than in males.

### 2.5. Prolactin-Positive Cells: Immunohistochemical Study and Densitometric Analysis

**Figure 11:**
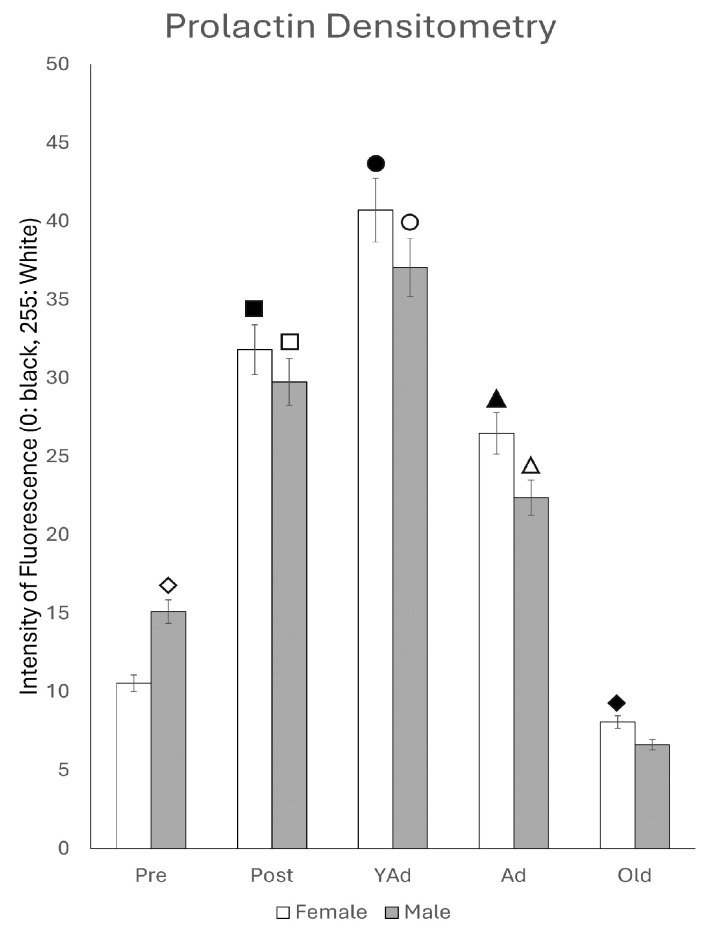
Densitometric values of the intensity of fluorescence for PRL (black = 0 and white = 255) found in the total of hippocampal structures studied (n: 5 animals per group studied). (Pre: Prepubertal animals, Post: Postpubertal animals, YAd: Young adult animals, Ad: Adult animals, and Old: Old animals). Two-tailed ANOVA results (Tukey’s test): ◇ *p* < 0.05 in relation to prepubertal females, ■ *p* < 0.01 relative to prepubertal females, ☐ *p* < 0.01 relative to prepubertal males, ● *p* < 0.05 in relation to postpubertal females, ○ *p* < 0.05 in relation to postpubertal males, ▲ *p* < 0.01 in relation to young adult females, △ *p* < 0.01 in relation to young adult males and *p* < 0.005 in relation to old males, and ◆ *p* < 0.005 in relation to adult females and *p* < 0.05 in relation to old males.

The densitometric analysis of the entire dorsal hippocampus, including Ammon’s horn and dentate gyrus, showed mean gray levels by sex and age stage that followed a pattern like that observed in the ELISA results (Figure 11). The highest values were found in postpubertal females and young adult animals. Except for prepubertal and young adult males, females showed significantly higher mean grey values than males at all other age stages. The degree of statistical significance is shown in Figure 11 and is described in detail below for each of the analyzed regions.

### 2.6. Prolactin-Positive Cells: Immunohistochemical Study and Regional Analysis

#### 2.6.1. CA1 Region

**Figure 12:**
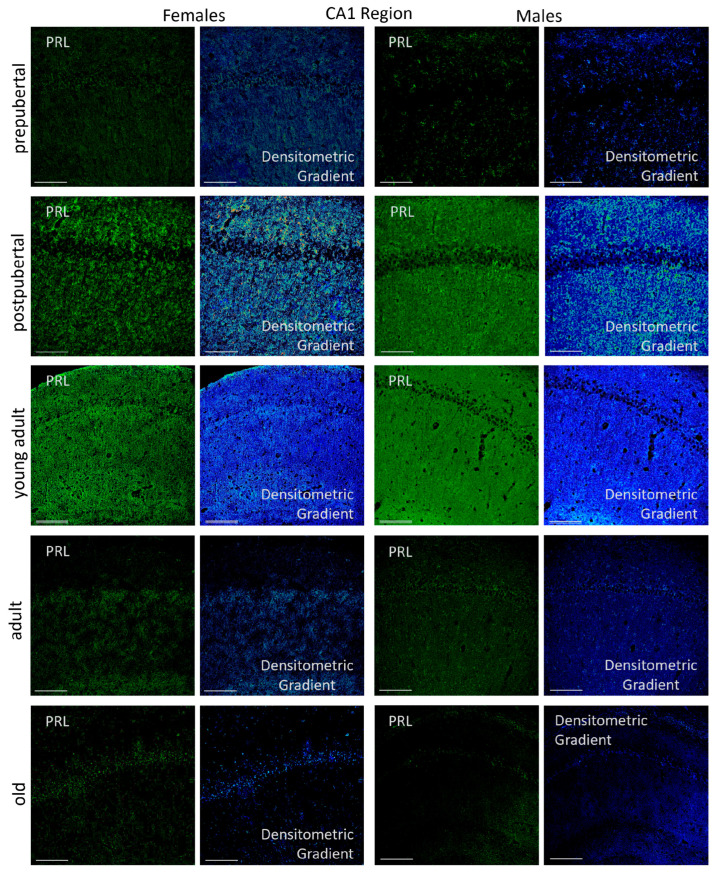
Confocal microphotographs of immunohistochemical localization of prolactin in the CA1 region of females and males at the different stages of life. In males, the distribution of prolactin in the CA1 region was similar, affecting all layers and with greater evidence in the pyramidal layer, although with reaction intensities that varied with age, the reaction being more evident in postpubertal males and young adults, who also presented a high intensity in the lacunous layer. In females, the location of the reaction varied with age, affecting all three layers in pre- and postpubertal females and young adults; in adult females, the reaction predominated in the radiatum layer and the lacunose layer; and in older females, it was mainly observed in the pyramidal layer. The images of the densitometric gradients highlight the reaction intensity in each of the layers across the different groups of animals studied. Scale bar: 116 µm, Double Scale bar: 200 µm.

The immunohistochemical reaction pattern observed in the CA1 region of female rats was consistent with the hormone levels detected via ELISA and the hybridization signal for *Prl* mRNA in this region (Figure 12). In prepubertal females, the reaction was mainly localized to neurons in the pyramidal layer. During the postpubertal periods, immunoreactivity became more prominent in the oriens layer, although an overall increase across all layers was evident, as shown by the densitometric gradient. Young adult females showed less reaction than postpubertal females, and the signal was mainly located in the pyramidal layer and the basal lacunous stratum to the radiatum layer. Immunostaining intensity declined in adult rats and was further reduced in old animals. In adults, the oriens layer remained the most affected layer, whereas in aged females, a punctiform reaction was observed in the neurons of the pyramidal layer and in some dendrites of the radiated layer. The distribution of prolactin in the CA1 region of male rats, unlike in females, presented an unexpected finding compared to the distribution of the hybridization signal. Specifically, the pattern and intensity of the reaction in postpubertal males resembled that of young adults. In prepubertal males, a punctate reaction was observed across all three layers of the CA1 region in an isolated and similar manner. Postpubertal and young adult males exhibited immunoreactivity in all three layers. As shown by the densitometric gradient, the reaction was greater in young adults and uniformly distributed across the CA1 region. In contrast, in postpubertal adults, the reaction was mainly located in the pyramidal layer and adjacent portions of the oriens and radiatum layers. In adult males, the reaction was mainly restricted to isolated pyramidal neurons. In old males, the reaction was limited to the pyramidal layer and isolated areas of the radiatum layer, being almost non-existent in the oriens layer. As described above, prolactin immunoreactivity was detected in all age groups of animals studied and in all strata of the CA1 region of the Cornu Ammonis analyzed (oriens, pyramidal and radiatum). However, densitometric analysis revealed statistically significant differences across age groups and between females and males.

**Figure 13:**
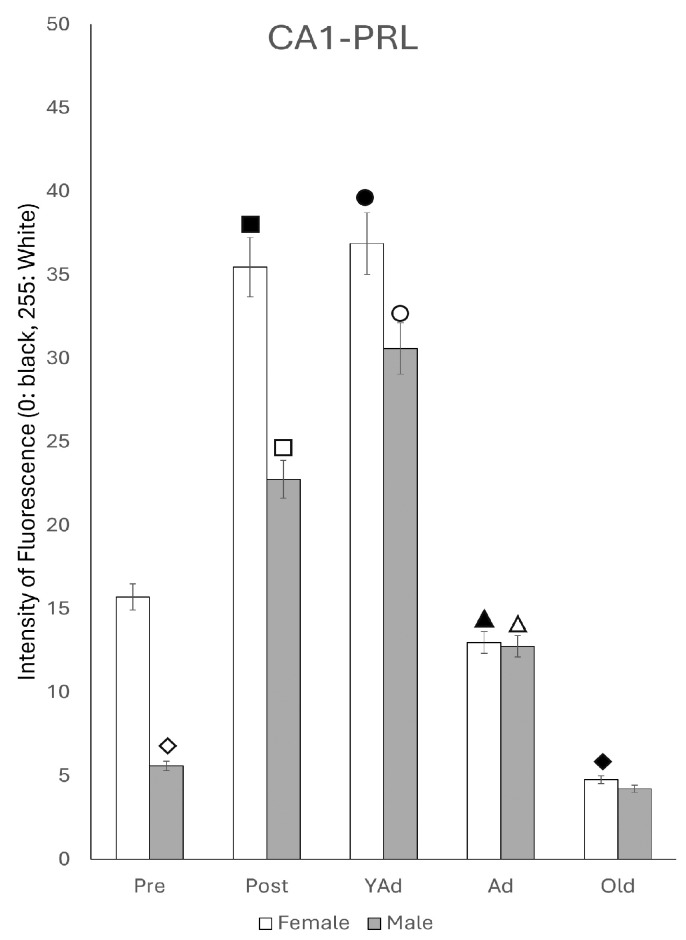
Densitometric values of the intensity of fluorescence for PRL (black = 0 and white = 255) found in the CA1 region (n: 5 animals per group studied). (Pre: prepubertal animals, Post: postpubertal animals, YAd: young adult animals, Ad: adult animals, and Old: old animals). Two-tailed ANOVA results (Tukey’s test): ◇ *p* < 0.01 in relation to prepubertal females, ■ *p* < 0.01 in relation to prepubertal and adult females and *p* < 0.005 in relation to old females, ☐ *p* < 0.005 in relation to prepubertal males and *p* < 0.01 in relation to postpubertal females, ● *p* < 0.01 in relation to prepubertal and adult females and *p* < 0.005 in relation to old females, ○ *p* < 0.05 in relation to postpubertal males and young adult females and *p* < 0.005 in relation to prepubertal males, ▲ *p* < 0.01 in relation to young adult female, △ *p* < 0.005 in relation to young adult males and *p* < 0.01 in relation to old males. ◆ *p* < 0.01 in relation to adult females.

Densitometric analysis (Figure 13) demonstrated that, in the CA1 region of female rats, the intensity of the reaction increased from the prepubertal to the postpubertal stage, remained stable in young adults, and then decrease in adulthood, and even more in old animals. Postpubertal and young adult females had a significantly higher reaction intensity than prepubertal (*p* < 0.01), adult (*p* < 0.01) and old females (*p* < 0.005). There were no significant differences between prepubertal and adult females or between postpubertal and young adult females. The lowest intensity was seen in old females (*p* < 0.01 vs. prepubertal females and adult females). In male rats, the intensity of the reaction increased progressively from the prepubertal stage to the young adult stage, then declined in adulthood and even more so in old males. The reaction intensity in prepubertal males was significantly lower than in age-matched females (*p* < 0.01). Postpubertal males presented a significantly more intense reaction than that of prepubertal males (*p* < 0.005), but significantly lower than that of females of the same age (*p* < 0.01). The intensity of reaction in young adult males was greater than in postpubertal males (*p* < 0.05) or prepubertal males (*p* < 0.001) and less than in young adult females (*p* < 0.05). The reaction decreased in adult males (*p* < 0.005 with respect to young adult males) without showing significant differences from adult females. Old males showed the lowest intensity of the reaction among all groups (*p* < 0.01 vs. adult males), although there were no significant differences in relation to old females or prepubertal males.

#### 2.6.2. CA3 Region

**Figure 14:**
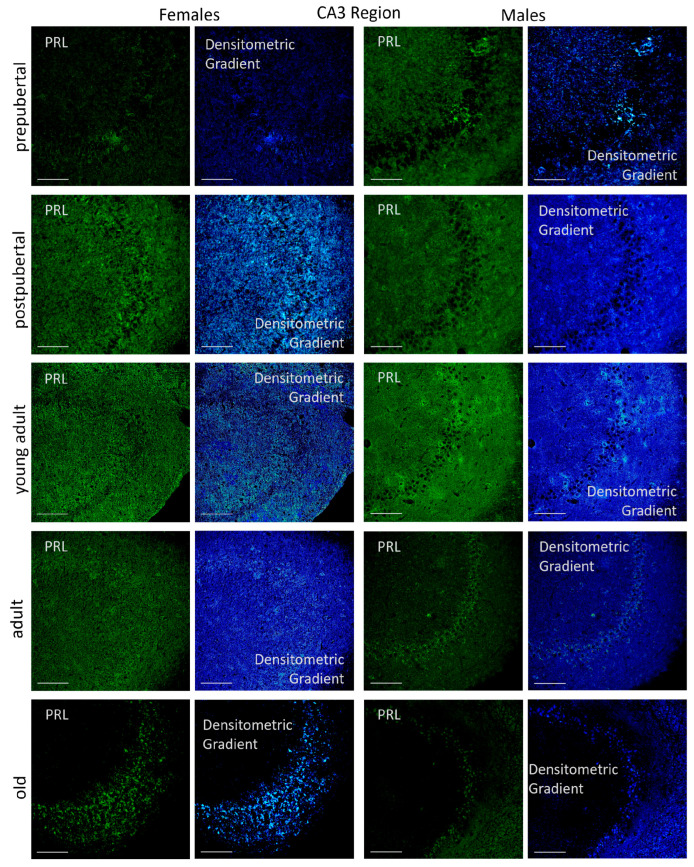
Confocal microphotographs of immunohistochemical localization of prolactin in the CA3 region of females and males at different stages of life. In prepubertal and old females and in adult males, prolactin was located almost exclusively in the pyramidal layer. In postpubertal and young adult animals, the reaction was distributed throughout all CA3 layers, although with an irregular distribution; some areas were more reactive than others within the same layer. The images of the densitometric gradients highlight the reaction intensity in each of the layers across the different groups of animals studied. Scale bar: 116 μm.

In both sexes, the reaction patterns in each layer studied, oriens, pyramidal and radiated in CA3, shared similar characteristics with those described for the CA1 reaction (Figure 14).

However, prolactin immunostaining was more intense in young adult females than in postpubertal females, with a greater signal observed in the oriens and radiatum layers than the pyramidal layer. In females, the fibrillar reaction observed in young adults decreased in adult females and disappeared in older females, who only had a reaction in the pyramidal layer. In males, the reaction was preferentially located in the pyramidal layer, although postpubertal males and young adults also had a fibrillar reaction in the oriens and radiatum layers. In contrast to females, old males showed reactivity not only in the oriens layer but also in the pyramidal layer of CA3.

**Figure 15:**
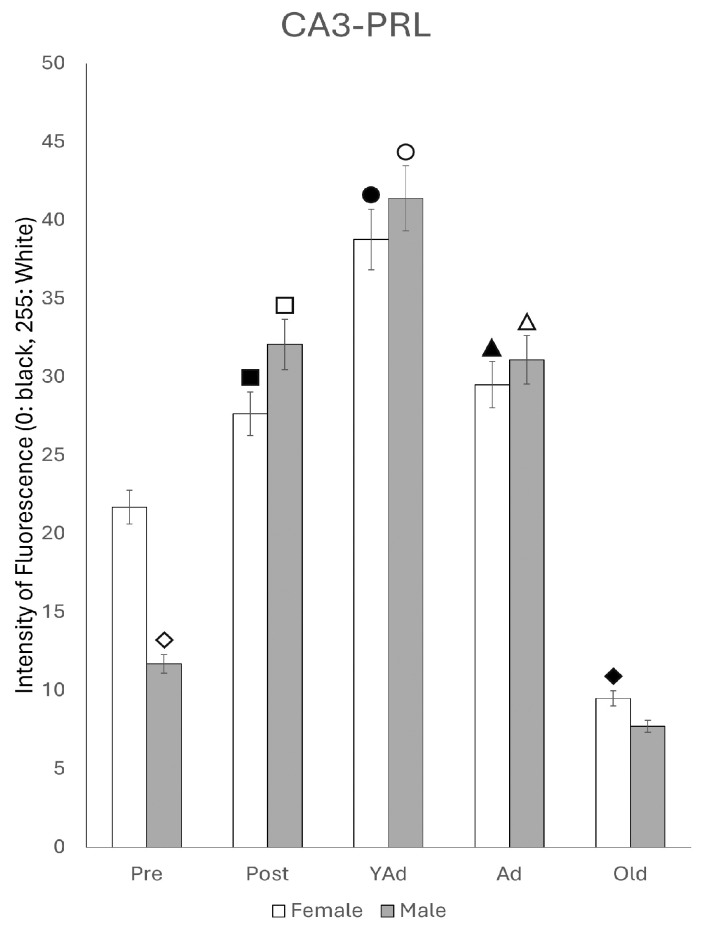
Densitometric values of the intensity of fluorescence for PRL (black = 0 and white = 255) found in the CA3 region (n: 5 animals per group studied). (Pre: prepubertal animals, Post: postpubertal animals, YAd: young adult animals, Ad: adult animals, and Old: old animals). Two-tailed ANOVA results (Tukey’s test): ◇ *p* < 0.01 in relation to prepubertal females, ■ *p* < 0.05 in relation to prepubertal females, ☐ *p* < 0.01 in relation to prepubertal males, ● *p* < 0.01 in relation to postpubertal females, ○ *p* < 0.01 in relation to postpubertal males and *p* < 0.005 in relation to prepubertal males, ▲ *p* < 0.01 in relation to young adult females, △ *p* < 0.01 in relation to young adult males and *p* < 0.005 in relation to old males, and ◆ *p* < 0.005 in relation to adult females and *p* < 0.05 in relation to old males.

Densitometric analysis of fluorescence intensity in the three layers of CA3 region revealed sexually dimorphic differences related to the age of the animals. These differences followed a similar pattern across all three layers (Figure 15). In female rats, intensity was low in prepubertal animals, increased significantly in postpubertal females (*p* < 0.05 vs. prepubertal females), and further increased in young adult females (*p* < 0.01 vs. postpubertal females). A significant decrease was observed in adult females (*p* < 0 vs. young adult females), followed by a slight further decline in old females (*p* < 0.005 vs. adult females). The reaction intensity in prepubertal males was lower than in females (*p* < 0.01), while in postpubertal, young adult and adult males, the values of medium gray observed were higher than those observed in females, but these differences did not reach statistical significance. In contrast, in old males, it was significantly lower (*p* < 0.05) than in females. Comparing age groups within males, postpubertal animals showed significantly higher intensity than prepubertal males (*p* < 0.01), and young adult males showed an additional increase (*p* < 0.01 vs. postpubertal males; *p* < 0.005 vs. prepubertal males). The intensity of fluorescence decreased in adult males compared to young adult males (*p* < 0.01), reaching similar values to those found in postpubertal males. Old males showed the lowest values in the intensity of fluorescence (*p* < 0.005 vs. adult males).

#### 2.6.3. Dentate Gyrus

**Figure 16:**
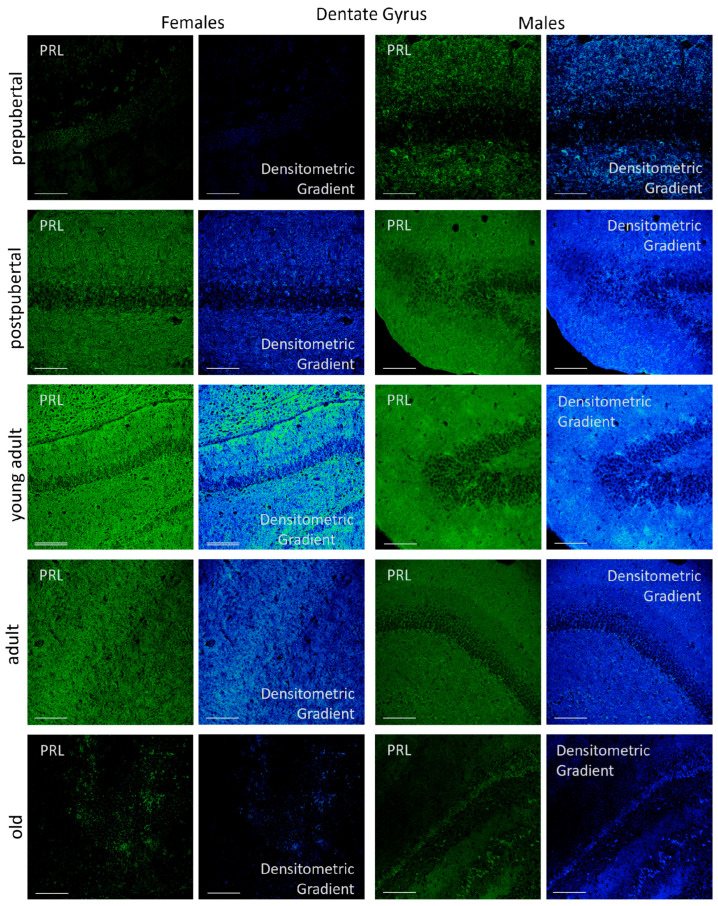
Confocal microphotographs of immunohistochemical localization of prolactin in the DG region of females and males at different stages of life. Although with age-related differences, prolactin was localized in the molecular, granular and polymorphic layers of the dentate gyrus in all the animals studied. Notably, the molecular layer of prepubertal males showed a stronger reaction compared to females of the same age. In general, throughout life, the intensity of the reaction increased until the young adult stage and then declined with the aging of the animals. The presence of positive subgranular cells was maintained until the stage of adult age. The images of the densitometric gradients highlight the reaction intensity in each of the layers across the different groups of animals studied. Scale bar: 116 µm, Double Scale bar: 200 µm.

In the dentate gyrus of prepubertal and old females, immunostaining was very weak. In contrast, males showed an evident reaction at the prepubertal stage and, as observed in postpubertal males, presented positive secondary neurons in the polymorphic layer (Figure 16). Another sex-related difference in the dentate gyrus was the presence of strongly positive subgranular cells in males. These cells were observed in both prepubertal and postpubertal males, with higher abundance in the latter, in which some of these cells began to invade the granular layer.

Young adult females reacted preferentially in the polymorphic layer close to the granular layer. Although subgranular cells were not detected, the granular layer showed alternating zones of positive cells and negative cells. Adult females showed a greater reaction in the molecular layer adjacent to the granular layer, and in older females, the reaction was limited to a few positive granular cells. Prepubertal males showed a reaction in the molecular and polymorphic layers, being very scarce in the granular layer, in which scattered reactive neurons appeared. Old males had a sparse reaction in the granular cells and a reactive band in the polymorphic layer, which was interposed between a non-reactive zone near the granular layer and another non-reactive zone near the CA4 neurons that invade the hilum of the dentate gyrus.

**Figure 17:**
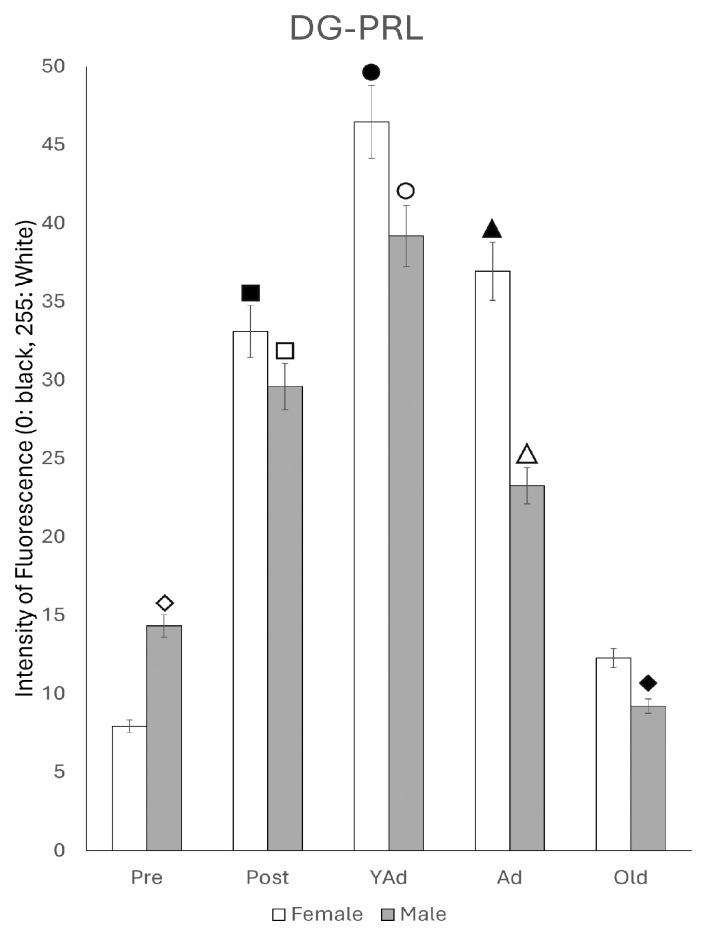
Densitometric values of the intensity of fluorescence for PRL (black = 0 and white = 255) found in the DG region (n: 5 animals per group of study). (Pre: prepubertal animals, Post: postpubertal animals, YAd: young adult animals, Ad: adult animals, and Old: old animals). Two-tailed ANOVA results (Tukey’s test): ◇ *p* < 0.01 in relation to prepubertal females, ■ *p* < 0.01 in relation to prepubertal females, ☐ *p* < 0.01 in relation to prepubertal males, ● *p* < 0.01 in relation to postpubertal females and *p* < 0.05 in relation to young adult males, ○ *p* < 0.05 in relation to postpubertal males, ▲ *p* < 0.05 in relation to young adult females, *p* < 0.01 in relation to adult males and *p* < 0.005 in relation to old females, △ *p* < 0.01 in relation to young adult males, and ◆ *p* < 0.01 in relation to adult males and *p* < 0.05 in relation to old females.

Densitometric analysis (Figure 17) of fluorescence intensity across the three layers of dentate gyrus region revealed some differences compared to those observed in Ammon’s horn. Nonetheless, age-dependent sexually dimorphic results were also evident. The intensity of fluorescence was significantly higher in prepubertal males than in age-matched females (*p* < 0.01). In postpubertal animals, intensity was higher than in prepubertal animals (*p* < 0.01, for both sexes), although no significant differences were observed between female and male animals. Again, fluorescence intensity increased in young adult animals in relation to postpubertal animals (*p* < 0.01 in females; *p* < 0.05 in males). Moreover, the intensity in young adult females was higher than in males (*p* < 0.05). In adult animals, fluorescence intensity declined compared to young adults (*p* < 0.05 in females; *p* < 0.01 in males), being higher in females than in males (*p* < 0.01). A significant and marked decrease in fluorescence intensity was observed in old animals compared to adult animals (*p* < 0.005 in females; *p* < 0.01 in males).

## 3. Discussion

It is well known that the characteristic parameters of biological rhythms can change with aging [23]. Controversial studies on the effects of aging on serum prolactin levels can be found in scientific literature, especially in humans [24,25].

Findings reported in rats differ from one study to another, mainly related to the variability in the age periods studied. Translating the results of other authors to the age periods of our study, there is sufficient evidence that in male rats, there is an increase in prolactin between puberty and adulthood [26,27], decreases in old males, which could be related to a decrease in pituitary lactotroph cell activity in older males [28] and that, in the female rat, serum prolactin increases with aging [27,28].

To our knowledge, no studies have reported prolactin levels in hippocampal lysates using ELISA or RIA. Some studies have used western blotting to explore prolactin in the brain [29], although these focused on its potential involvement in Alzheimer’s disease associated with diabetes. This is the first experimental study in rats to analyze the hippocampal prolactin system across the lifespan by combining hormonal determinations via ELISA, characterization of hippocampal prolactin with immunohistochemistry, and expression of hippocampal *Prl* mRNA via qPCR and in situ hybridization.

In our study, using specific probes for *Prl* mRNA, we were able to demonstrate the presence of hippocampal *Prl* mRNA. This is the first evidence of this mRNA using in situ hybridization in rats. Because animals were perfused and hematic rests were removed, the results obtained in our study cannot be attributed to hematic contamination.

### 3.1. Circulating Versus Hippocampal Prolactin: Differential Regulation Across Life Stages

In general, the findings of our study are in line with those described by other authors, mainly regarding higher serum prolactin levels in females compared to males [25,30]. It is also important to note that, in the animals in our study, the possibility of prolactinomas that are known to develop spontaneously with aging [31,32,33] was ruled out. Although human studies have shown that blood prolactin levels are higher in women than in men, they have not described age-related variations [25]. However, in rats, an increase in serum prolactin levels has been demonstrated in female rats and has been linked to a disruption in the regulatory mechanisms of pituitary prolactin secretion, especially disruptions in dopaminergic and GABAergic regulations [26].

Our study is the first to analyze age-related changes in hippocampal prolactin levels in hippocampal lysates using ELISA. Analysis of the results obtained shows that prolactin levels were higher in females than in males across all age groups studied, suggesting sexual dimorphism that could be related to sex steroid levels, mainly estrogens. Our results also indicate that *r*-value in the correlation study was negative in females and, although the *r*-value was positive in males, it did not reach statistical significance, indicating that serum prolactin and hippocampal concentrations are not totally directly related. Because the results obtained from ELISA, qPCR and densitometric analyses of immunoreactivity to prolactin and *Prl* mRNA hybridization showed some age-related differences, the most likely conclusion is that, under several age-related conditions, part of hippocampal prolactin passes from the bloodstream. Without rejecting this possibility and considering the lack of correlation between serum and hippocampal prolactin levels, our data from immunohistochemistry, qPCR, and in situ hybridization further suggest that prolactin is synthesized within the hippocampus and dentate gyrus, which occurs in neurons and is modified in relation to age and sex.

### 3.2. Evidence for Local Synthesis of Prolactin in the Hippocampus: Technical and Conceptual Validation

The precise mechanism through which biologically active prolactin may access the brain remains unclear. There have been numerous reports on both prolactin and *Prl* mRNA expression in brain tissue. However, there are many controversies from one study to another that mainly deal with the origin of detectable prolactin in the brain and the specificity of what was found. One explanation for this might be that these studies detected pituitary prolactin after transported from the periphery. However, in at least one of these studies [15], prolactin-like immunoreactivity was not eliminated by hypophysectomy, suggesting that it was unlikely to be of pituitary origin.

Because prolactin is a large protein (M.W.: 23 kDa) that can dimerize several times, it would be expected to be excluded from the brain by the existence of specialized tight junctions between vascular endothelial cells in the brain: the blood–brain barrier [34]. However, circulating hormones less than 40 kDa in size can pass from the bloodstream to the brain in places where fenestrated capillaries exist, such as the median eminence and the ventromedial portion of the arcuate capillary nucleus [35,36]; however, the presence of fenestrated capillaries in the hippocampus has not been described.

Within the central nervous system, although with discrepancies among authors, the possibility of synthesizing prolactin that would act locally has also been described [37]. The possibility of prolactin production in the brain started when the hormone could still be detected in the brains of animals after removal of the pituitary gland [15].

In this study, the presence of prolactin in the dentate gyrus and hippocampus—CA1 and CA3—was demonstrated by means of immunocytochemistry and ELISA in a similar way as previously described by other authors [15,38].

Using qPCR, *Prl* mRNA was detected in several regions of the brain, such as the pons-medulla, hypothalamus, amygdala, thalamus and choroid plexus in rodents [16,39,40,41,42,43,44].

Using western blotting and in situ hybridization, the presence of prolactin and *Prl* mRNA in the brain of sheep was confirmed and was localized in the medial preoptic area, periventricular preoptic nucleus, bed nucleus of the stria terminals and ventral paraventricular nuclei [18]. Most of these studies were performed on female animals during diestrus, pregnancy, lactation, and perinatal pups. There has been no confirmation using qPCR or in situ hybridization for the hippocampal synthesis of prolactin in the past.

This could be important because the extreme sensitivity of RT-PCR means there is potential for *Prl* mRNA to be detected from contamination by lactotrophs in the tuberal region of the pituitary stalk [45] or from circulating immune cells present within the tissue that may express prolactin [46,47].

The possibility of contamination from the pituitary pars tuberalis can be accepted when the structure to be studied is the hypothalamus, as it is very common for the glandular portion to be attached through the meninges to the basal face of the median eminence of the hypothalamus. In our study, this possibility does not occur because, as explained in the chapter on material and methods, a careful dissection of the rat hippocampus was made under a microscope, which, on the other hand, is too far from the pituitary gland and median eminence to leave pituitary cells adherent. Moreover, during hippocampal dissection, once the lateral ventricle is accessed, the first step is to remove the choroid plexus. Although there may be hematic rests in intrahippocampal vessels, they are not enough to suggest that there is contamination by immune cells that falsify the results obtained with qPCR, which, on the other hand, have been confirmed via in situ hybridization. In our study, analyses have been carried out using different primers, based on sequences published by other authors [40,48], for the *Prl* mRNA, and comparing the results, it was concluded that the primers used in the study were the ones that gave the most reliable results.

Some laboratories have questioned the immunoreactivity of prolactin in the brain since the antibody used could cross-react with opiomelanocortin [49], which might account for some of the observations. In contrast, in this study, substitution of primary antibody and pre-absorption of the primary antibody used in immunohistochemistry with prolactin was performed. As a result, no cells appeared positive, and with ACTH, the reaction was not modified. In addition, since the pars intermedia is very rich in opiomelanocortin, the pattern of the reaction between the two positive pituitary controls, one for prolactin and the other for ACTH in the pars intermedia, were contrasted, resulting in these patterns being totally different. Moreover, light- and electron-microscopic immunocytochemical studies have demonstrated that although corticotropin (ACTH 17-39)-immunoreactive fibers could be detected in several regions found to contain prolactin fibers, the distribution and organization of both fiber types clearly differ in numerous brain regions, and the regions containing the corresponding perikarya do not overlap [50].

### 3.3. Possible Functional Implications: From Neurogenesis to Hippocampal Regulation

More than 300 different biological functions have been described for prolactin, some of which are related to the central nervous system, including the stimulation of neurogenesis, modulation of stress responses, reduction of anxiety, transport of calcium and regulation of the immune system [1,2,3]. However, most of these studies were carried out in states of hyperprolactinemia or after exogenous administration of the hormone, which makes it difficult to determine whether these results are physiological or pharmacological. The literature includes different studies that relate prolactin to maternal behavior, lactation, pregnancy and hyperprolactinemic physiological states [51,52,53,54,55,56]. Exogenous administration of prolactin has been linked to similar neurogenic processes in adults, particularly involving the subventricular zone [1] and the hippocampal subgranular zone [5], where it has also been shown to exert neuroprotective effects [5,6,7,48,57,58,59].

The presence of prolactin and *Prl* mRNA in the brain and hippocampus has been described via RIA, immunocytochemistry, RT-PCR and RT-qPCR [15,16,17,18,19] and has been associated with neurodevelopment [60], neurogenesis, cell proliferation, neuroprotection [7,57,58], neuroplasticity and dendritic remodeling during pregnancy and the postpartum period [61,62]. Prolactin is associated with neurogenesis in the dentate gyrus [22,59,63,64,65]. The subgranular zone of dentate gyrus maintains proliferation capacity in adulthood [22,59]. Neurogenesis in the dentate gyrus has been directly related to learning, memory and spatial orientation [21]. In the hippocampus of adult mice, prolactin may be necessary for learning and for enhancing long-term memory consolidation [22,66,67,68]. The presence of positivity to prolactin in the subgranular cells in the dentate gyrus that we observed in our study suggests that prolactin could have a neurogenic paracrine effect, mainly from the prepubertal stage to the young adult stage.

The synthesis of de novo prolactin in the hippocampal regions observed in our study, obtained via qPCR and in situ hybridization, suggests the possibility that prolactin could play an auto-paracrine regulatory role in the hippocampus, which undergoes variations in the aging of the animal, and it is sexually dimorphic. Because prolactin has a potentially neuroprotective effect [5,6,7,48,57,58,59], it is possible that the increases in hippocampal prolactin detected in old animals, mainly in females, may be related to this physiological function.

### 3.4. From Undetected to Demonstrated: How Region-Specific Focus and Multiple Techniques Revealed Hippocampal Prolactin Expression

Most studies analyzing the presence of prolactin in the brain via immunocytochemistry have been conducted by studying the hypothalamus or choroid plexuses. The original observation of immunoreactive-prolactin in the hypothalamus of rats [69] has been supported by several groups [15,70,71,72,73,74]. Prolactin has also been detected via push-pull perfusions within the brain, with changing levels associated with conditions such as lactation [43,44]. Different studies have detected the presence of extrahypothalamic cerebral prolactin and its independence from pituitary prolactin in areas such as cerebellum, thalamus, brainstem (pons-medulla), hippocampus, cerebral cortex and caudate [15,16,50,75], concluding that the transcriptional regulation of PRL in the brain is different from that in the anterior pituitary. In the cerebellum, transcriptomic analysis has detected a decrease in *Prl* gene expression in relation to aging [76].

An important finding in our study is the modifications in *Prl* mRNA expression in the hippocampus and dentate gyrus during aging. Usually, the lowest signal was found in adults and old animals, and compared to pre- and/or postpubertal animals, the signal was almost non-existent in some layers of certain regions. This could explain the controversy that *Prl* mRNA may or may not be found via in situ hybridization, since false negatives could be obtained if the studies were carried out on animals older than 3 months. In our study, variations in *Prl* mRNA expression observed via qPCR and in situ hybridization across different age stages and sex analyzed are concordant with the findings obtained via ELISA and immunocytochemistry, so that the decrease in the hybridization signal, as well as the decrease in the expression of *Prl* mRNA found after qPCR in the same periods, could justify the decrease observed when the protein was analyzed.

The identification of the hippocampus as a site of prolactin synthesis raises key questions about its functional role in the brain. Prolactin production in the central nervous system has been reported in certain circumstances such as brain development [22], brain’s response to injury [48,77,78,79,80] and in vitro studies of hypothalamic fragments or glial cells in culture [70,81,82].

The knowledge gap leads us to formulate other questions: “How is presence of prolactin and expression of *Prl* mRNA in the hippocampal and dentate gyrus regulated?” and “What accounts for the observed pre- and postpubertal changes, as well as the decrease observed in adult and old animals?” These questions, raised by our results, represent a new challenge that needs to be studied in the future.

In summary, our findings suggest that the hippocampal prolactin system is constituted by two different sources of prolactin, the local synthesis of the hormone and its uptake from the blood. As previously described for the synthesis of prolactin receptor isoforms in the hippocampus from our laboratory [83], the synthesis and presence of the hormone in the hippocampus is significantly modified throughout life and is also sexually dimorphic.

Considering the biological effects described for prolactin in the brain, the fall in prolactin levels and its synthesis in the hippocampus, together with that of its receptors, could be involved in brain aging.

## 4. Materials and Methods

### 4.1. Animals and Groups to Study

A total of 100 Wistar rats (*Rattus norvergicus*) were used for this study. Fifty of them were used for histological analysis, and the other 50 were used for molecular analysis. Rats that developed pathologies due to aging, like diabetes or tumors, mainly prolactinomas, were discarded at the time of sacrifice. The experimental groups were defined as follows: prepubertal group: 4 weeks old; already separated from the mother and not lactating but had not yet reached puberty. Postpubertal group: 6 weeks old; reached puberty within 5 days before the day of sacrifice. Young Adult: 4–5 months old, virgin and not exposed to mating. Adult: 9–10 months old; virgin and not exposed to mating. Old: 15+ months; virgin, not exposed to mating and could not reproduce anymore. Each group consisted of 10 animals per sex. Of these, 5 were allocated for histological analyses and 5 for molecular studies. Every female rat (from postpubertal to adult age) was sacrificed during the estrus stage of the estrous cycle. All animals were kept in standard conditions for experimental rats, with a light–dark cycle of 12–12 h and free access to food and water resources *ad libitum*. All methods and procedures involved in this experiment were approved by the Ethics Committee of the University of Salamanca and were conducted in accordance with the animal care guidelines of the European Common Council (86/609/EEC) and Spanish regulations (Royal Decree 1201/2005), making significant efforts to minimize both suffering and the number of animals used. All animal experiments comply with the ARRIVE guidelines and the EU Directive 2010/63/EU on the protection of animals used for scientific purposes.

### 4.2. Animal Sacrifice and Sample Collection

For histological studies, animals were sedated with equitesin, a mixed solution of pentobarbital, chloral hydrate, ethanol, propylene glycol, Mg sulphate and bidistilled water. After opening their thorax, they were killed via trans-cardiac perfusion by substitution of the blood with heparinized saline serum (0.9% NaCl) and then perfused with paraformaldehyde (4%). By careful dissection, brains were taken out of their skull and kept in paraformaldehyde (4%) for the next 24 h. To preserve the tissues for freezing, brains were submerged in increasing solutions of sucrose in Phosphate-buffered saline (PBS 0.1 M, 0.9% NaCl, pH 7.4) and then frozen, and serial sections of 20 µm thickness were obtained with a Microm^®^ cryostat (Microm Internaitonal GmbH, Wallford, Germany). The sections were introduced in wells with PBS and then fixed to be placed in coated microscope slides.

For analytical and molecular studies, animals were sedated with equitesin and sacrificed by decapitation. Blood samples were taken from the neck, and serum was obtained via centrifugation. Samples were stored at −80 °C until the day of the ELISA. By careful dissection, brains were taken out of their skull and kept in synthetic cerebrospinal fluid for dissection of hippocampus (gasified aqueous solution of 3 mM KCl, 1.25 mM NaH_2_PO_4_, 28 mM NaHCO_3_, 14.56 g of sucrose, 0.36 g of Dextrose, 2 mL of 1 mM MgCl_2_, 100 mL of 1 M CaCl_2_ and 1 mL of 0.6 M sodium pyruvate) using a protocol designed specifically for this study. To obtain the cerebral block containing the hippocampus, the procedure previously described in our laboratory was followed [83]. According to The Rat Brain in stereotaxic coordinates [84], two frontal cuts were made: one cephalic cut at the intersection of the fornix column with the corpus callosum, and one caudal cut in the cephalic part of the superior colliculus. In a third step, by dissecting below the corpus callosum toward the lateral ventricle, the striatum and thalamus were moved medially. Finally, through careful dissection, the hippocampus was separated from the rest of the cortex following its dorsolateral limit, which is given by the corpus callosum, until it was completely separated. Once isolated, the hippocampus was frozen by immersion in liquid nitrogen and stored in a freezer at −80 °C.

### 4.3. ELISA Analysis Technique

To determine serum and hippocampal prolactin levels, a rat prolactin ELISA kit (Invitrogen^®^ ERA50EB provided by Thermo Fisher Scientific, Waltham, MA, USA) was used according to the manufacturer’s instructions.

### 4.4. Immunostaining for Prolactin

After drying the frozen slides, they were washed in TBS (0.05 M Trizma base, 0.8% NaCl, pH 7.5. Sigma-Aldrich, St. Louis, MO, USA). Blocking was performed with Bovine Albumin Serum (BSA Sigma-Aldrich. Diluted at 1/30 in TBS). The samples were incubated overnight at 4 °C in a humidity chamber with rabbit primary antibody for human prolactin (Dako, A0269/lot. 076, reactive for human, rat and mouse, Agilient DAKO Santa Clara, CA, USA) diluted 1/250 in TBS. After 2 washes in TBS for 5 min at room temperature, the samples were incubated for 3 h at room temperature in a dark chamber with goat anti-rabbit conjugated with AlexaFluor 488 (Abcam, Cambridge, UK. ab150077) diluted 1/800 in TBS. After washing the slides on TBS three times for 5 min each, the samples were incubated with Hoechst 33342 (Cayman Chemical, Ann Arbor, MI, USA. 15547) diluted 1/1500 for 15 min at room temperature. After 2 washes in TBS and 1 wash in distilled water, the slides were covered with Fluoromont (Sigma-Aldrich) medium and sealed.

The anti-prolactin antibody has been used in previous studies on the pituitary gland [85]. To test the specificity of the antibody, preabsorption tests with prolactin and tests replacing the specific serum with normal non-immune rabbit serum abolished the reaction. Using ELISA, the specificity of goat anti-rabbit IgG was lower than 1% for rat and mouse IgG and 100% for rabbit IgG. Preabsorption with ACTH did not modify the immunohistochemical reaction.

### 4.5. Immunostaining for Pituitary Prolactin and ACTH (Opiomelanocortin)

**Figure 18:**
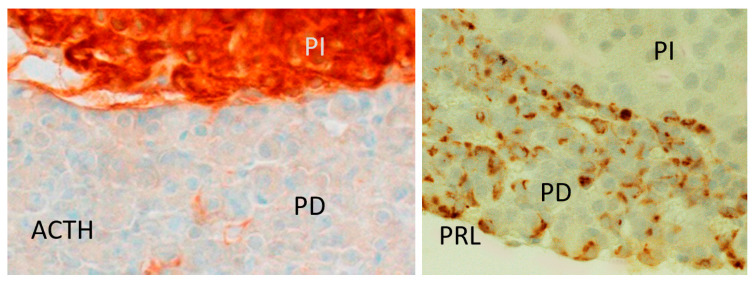
Microphotographs show the results after immunohistochemistry for prolactin (PRL) and adrenocorticotropin hormone (ACTH). As seen in the images, the reaction for prolactin was located only in the pars distalis (PD) of the pituitary gland, while the reaction for ACTH was very intense in the pars intermedia (PI) and in some scattered glandular cells of the pars distalis. The reaction patterns and intraglandular locations of both hormones were totally different.

A positive control for prolactin and a comparison between the reaction pattern of the antibody used for the prolactin study and that of the reaction for opiomelanocortin were carried out on 7 mm rat pituitary sections obtained from untreated adult female rats, sourced from the laboratory’s specimen archive. After deparaffinizing and rehydrating the slices, endogenous peroxidase was inhibited in a methanol−3% hydrogen peroxide solution (200 mL/20 mL, *v*/*v*) for 30 min, followed by two 5-min TBS (0.05 M Trizma base Sigma-Aldrich, 0.8% NaCl, pH 7.5). Blocking was performed with Bovine Albumin Serum (BSA Sigma-Aldrich, diluted 1/30 in TBS). The samples were incubated overnight at 4 °C in a humidity chamber with rabbit primary antibody for prolactin (Dako, A0269/lot. 076) diluted 1/250 in TBS or ACTH (Dako, M3501) diluted 1/500 in TBS. After 2 washes in TBS for 5 min each at room temperature, the samples were incubated for 45 min at room temperature in a humidity chamber with biotinylated goat anti-rabbit antibody (Abcam^®^, ab150077) diluted 1/800 in TBS. After 2 washes in TBS for 5 min each, the samples were incubated with Streptavidin–Peroxidase complex (Abcam, 15547) diluted 1/1500 for 45 min at room temperature. After 2 washes in TBS, the reaction was developed with 3-3’ diaminobenzidine (25 mg /100 mL of TB (0.05 M Trizma base, Sigma-Aldrich, pH 7.4)) plus 150 mL of 3% hydrogen peroxide under microscopic control, and then washed in distilled water. The slides were counterstained with Mayer’s hematoxylin, washed in water and covered with Aquaplast (Sigma-Aldrich). The results are shown in Figure 18. The following negative controls were used for prolactin immunohistochemistry: substitution of the primary antibody with TBS; substitution of secondary antibody with TBS; preabsorption of the primary antibody with human prolactin or ACTH (Sigma-Aldrich) at 10 ng/100 mL overnight. Positive pituitary control and contrasting immunostaining patterns between prolactin and ACTH (opiomelanocortin) in the pituitary pars intermedia (PI in Figure 18) were included.

### 4.6. In Situ Hybridization

After drying the frozen slides, they were submerged in acetylated buffer for 10 min. Then, they were washed with distilled water and dried in a stove at 37 °C for 20 min. Pre-hybridization was made with Omnibuffer (Wak-Chemie Medical GmbH, Steinbach (Taunus), Germany) in the HybAid Omnislide thermocycler (Thermo Fisher Scientific) with a humidity chamber for 1 h at 37 °C. Hybridization was performed in the same chamber at 37 °C overnight with the following 5′ biotinylated probes:

Sense probe for Rat PRL: {Btn}CATTACAACTTTCAGCACATGCTTAAGT.
Antisense probe for Rat PRL: {Btn}GTAATGTTGAAAGTCGTGTACGAATTCA.

After hybridization, slides were submerged two times in the HybAid Omnislide washing module (Thermo Fisher Scientific) with astringent washes using 0.1% SSC buffer (sodium citrate solution) at 37 °C for 5 min each. They were then again washed one last time with the same buffer at room temperature for another 5 min. To avoid false positive detection, after hybridization and astringent washes, the samples were treated with RNase. Biotin was detected via immunohistochemistry. For this purpose, the samples were washed twice in TBS for 5 min at room temperature. Blocking was performed with BSA for 30 min and then incubated overnight at 4 °C in a humidity chamber with a primary mouse antibody Anti-Biotin (Dako, M0743) diluted 1/350 in TBS. After washing on TBS twice for 5 min at room temperature, the samples were incubated for 3 h at room temperature, with goat anti-mouse antibody conjugated with AlexaFluor 488 (Abcam. Ab150113) diluted 1/800 in TBS. The samples were then washed twice with TBS and Hoechst 33342 (Cayman Chemical, 15547) at a dilution of 1/1500 for 15 min. Finally, after washing with TBS twice and leaving the slides for another 5 min in distilled water, Fluoromont (Sigma-Aldrich) was applied, and the slides was covered and sealed.

**Figure 19:**
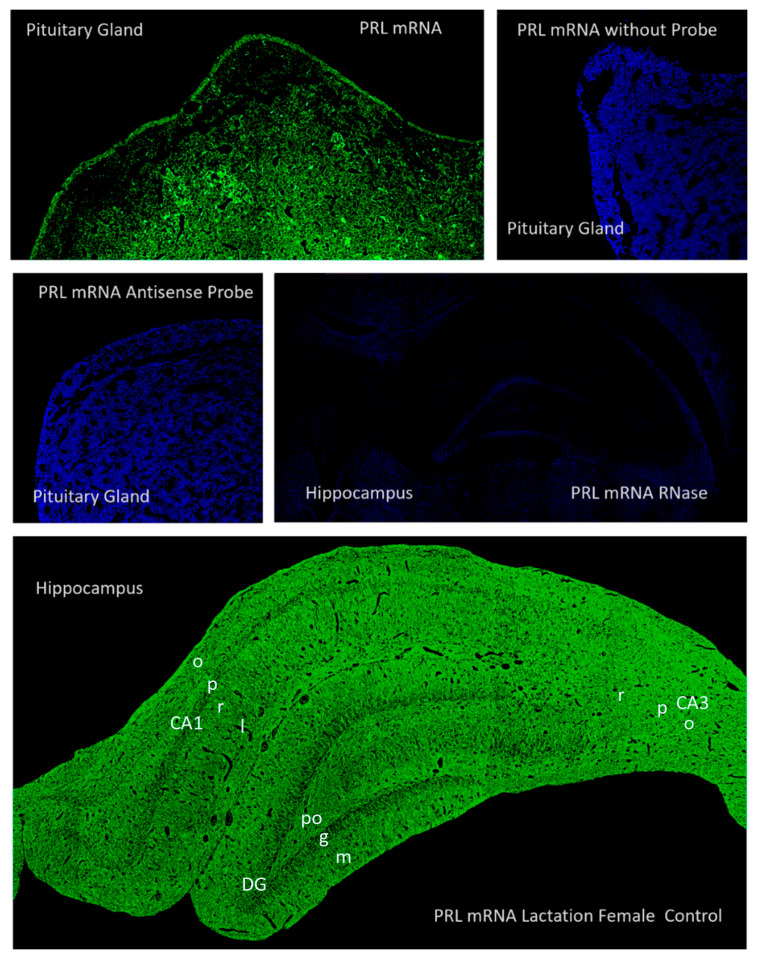
Microphotographs showing the results obtained in the control tests for in situ hybridization of *Prl* mRNA. a: Positive control for *Prl* mRNA in the anterior pituitary gland. b: Negative control for *Prl* mRNA in the pituitary gland after in situ hybridization without probe. c: Negative control for *Prl* mRNA in the pituitary gland after in situ hybridization following previous incubation of the sense probe with the antisense probe. d: Negative control for *Prl* mRNA in the hippocampus previously incubated with RNase. e: Positive control in the hippocampus of a lactating female rat. (o: oriens layer, p: pyramidal layer, r: radiate layer, l: Lacunous stratum, m: molecular layer, g: granular layer, po: polymorphic layer. CA1: cornus amonii region 1, CA3: cornus amonii region 1 and DG: dentante gyrus). Blue color: Hoescht nuclear staining, Green color: immunofluorescence.

Hybridization positive controls for rat PRL mRNA were performed on pituitary gland and hippocampus sections obtained from female rats (48 h of lactation), and negative controls included similar sections treated with the same quantity of sense and antisense probes, substitution of probe with omnibuffer, and after pre-treatment of tissue with RNase enzyme. Figure 19 shows some of the results obtained for positive and negative controls of PRL mRNA in situ hybridization in the hippocampus and pituitary gland.

### 4.7. Image Analysis

Images were acquired using a confocal microscope Stellaris (Leica Microsystems GmbH, Wetzlar, Germany) at ×20 magnification and a resolution of 2048 × 2048 pixels. The following hippocampal regions were photographed: CA1, CA3 and DG. A Z-Stack was performed, and the exported images were the max exposure composition of all the photos taken along the Z axis at the same X and Y position. The same pinhole, gain, brightness and contrast were used to obtain the digital microphotographs.

Using ImageJ 1.54p (National Institutes of Health, Bethesda, MD, USA), densitometric analysis of the digital images was carried out. From 10 microphotographs per animal, fluorescence intensity for prolactin immunohistochemistry and prolactin in situ hybridization was analyzed. Mean grey values were obtained for the oriens, pyramidal and radiate layers in the CA1 and CA3 regions of Cornus Amonii, and the grey mean values for the molecular, granulate and polymorphic layers in DG were obtained.

### 4.8. Immunofluorescence Gradient Python Script

Immunofluorescence images were processed using a custom Python script to generate intensity gradient images and determine the densitometric values for every pixel. The script assigns and intensity values that range from 0 to 255 in the original image and replaces that pixel on a scale where cold colors represent low intensity values and warm colors are higher values, resulting in an image where the most intense pixels can be easily detected. The main difference between this gradient and others used in industry is that the purple color has been removed from the scale, as it usually appears between blue and teal colors, and we consider purple a warm color, confusing the interpretation of the images. The script was programmed in Python 3.89.6 by Julian Happel [86]. IF-Grad: Fluorescent image gradient converter (v 0.1.2) free computer software (https://github.com/chaotix1992/IF-Grad, accessed on 17 November 2023).

### 4.9. q-PCR Technique

Messenger RNA Extraction and Complementary DNA Synthesis.

After hippocampus dissection, the tissue was immediately frozen in liquid nitrogen and stored at −80 °C till RNA isolation. Isolation was performed using RNeasy Mini and QIAshredder Kits (Qiagen N.V., Venlo, The Netherlands. #74104 and #79654) according to the manufacturer’s instructions. RNA was stored at −80 °C and used for cDNA synthesis using random primers and MultiScribe Reverse Transcriptase (Thermo Fisher Scientific, High-capacity RNA-to-cDNA kit #4387406), with the following mix per sample: 10 μL of 2× buffer mix, 1 μL of 20× enzyme, up to 2 μg or up to 9 μL of RNA sample, and nuclease-free water to a total of 20 μL, in a thermal cycler with the following program settings: 10 min at 25 °C, 120 min at 37 °C and 5 min at 85 °C. The cDNA obtained was stored at −20 °C till the qPCR experiment.

Realtime quantitative PCR (q-PCR) was performed using gene-specific primers spanning exon−exon junctions in the target mRNA, AmpliTaq Gold DNA polymerase, and a SYBR Green I detection kit on a QuantStudio 3 System (Thermo Fisher Scientific). For a total volume 20 µL per well, 2 µL of cDNA previously diluted to 20 ng/μL, 10 µL of SYBR Select Master Mix (Thermo Fisher Scientific, 4472908), 0.1 µL of forward primer previously diluted at 20 µM, 0.1 µL of reverse primer previously diluted at 20 µM, and 7.8 µL of double-distilled water were mixed.

The qPCR protocol began with a hold was carried out in 2 steps at 50 °C and 95 °C for 2 and 10 min, respectively. Forty PCR cycles at 95 °C for 15 s and 60 °C for 1 min were performed. Melting curve was carried out by slow temperature transitions in 3 steps: heating at 95 °C for 15 s, cooling to 60°C for 1 min (ramp rates 1.6 °C/s), and heating again to 95 °C for 5 s (ramp rate 0.15 °C/s). mRNA abundance of the target genes in each sample was normalized based on its Hprt-1 content.

Primers were first tested to determine whether they were optimal for our experiments. Primers for *Prl* were tested in the pituitary gland and the hippocampus. The ratio was calculated using *Hprt1* as the housekeeping gene (HKG), whose expression was stable in all samples, using the following formula:

Ratio (%) = 2^(−ΔCt)^ × 100, where ΔCt = Average target Ct−Average HKG Ct

Primer sequences 5′-3′:

PRL forward CCACCTAGTCCAGTTATTAGTTGA.

PRL reverse CCCTAGCTACTCCTGAAGACA.

HPRT1 forward CCCAGCGTCGTGATTAGCGAT.

HPRT1 reverse CGAGCAAGTCTTTCAGTCCTGTCCATA.

### 4.10. Statistical Analysis

All data were analyzed using GraphPad Prism (8.4.3 version).

Descriptive statistical analysis was performed, including the calculation of minimum, maximum, range, arithmetic mean, standard deviation and standard error of the mean. Inferential analysis was conducted using two-way analysis of variance (ANOVA). The significance of multiple comparisons was checked using Tukey’s test. The confidence interval was 95% where *p*-value was significant if *p* < 0.05, and very significant if *p* < 0.01 or more.

## Figures and Tables

**Table 1:** Values obtained from Pearson’s *r* correlation analysis, showing that there was no correlation between serum and hippocampal levels in either sex or age groups.

Pearson *r*	Males	Females
*r*	0.2402	−0.09306
95% confidence interval	−0.8147 to 0.9262	−0.9013 to 0.8598
R squared	0.05769	0.00888661
*p* value		
*p* (two-tailed)	0.6971	0.8817
*p* value summary	No Significant	No Significant
Significant? (alpha = 0.05)	No	No

## Data Availability

The data presented in this study are available upon reasonable request from the corresponding author.

## Abbreviations

The following abbreviations are used in this manuscript:

ACTH	Adrenocorticotropic hormone
Ad	Adult animals
ANOVA	Analysis of variance
CA1	Cornus Amonii (Ammon’s horn) 1 region
CA3	Cornus Amonii (Ammon’s horn) 3 region
CA4	Cornus Amonii (Ammon’s horn) 4 region
CaCl_2_	Calcium chloride
cDNA	Complementary deoxyribonucleic acid
CSF	Cerebrospinal fluid
DG	Dentate Gyrus
DNA	Deoxyribonucleic acid
ELISA	Enzyme-Linked ImmunoSorbent Assay
HPRT1	Hypoxanthine phosphoribosyl transferase 1, housekeeping gene
KCl	Potassium chloride
kDa	Kilo Daltons
M.W.	Molecular weight
MgCl_2_	Magnesium chloride
mRNA	Messenger ribonucleic acid
NaCl	Sodium chloride
NaH_2_PO_4_	Sodium phosphate
NaHCO_3_	Sodium bicarbonate
PBS	Phosphate buffer saline
PCR	Polymerase chain reaction
PD	Hypophyseal pars distalis
PI	Hypophyseal pars intermedia
Post	Postpubertal animals,
Pre	Prepubertal animals,
PRL	Prolactin hormone
*Prl* mRNA	Mesenger ribonucleic acid for prolactin
qPCR	quantitative polymerase chain reaction
RIA	Radio immune assay
RNA	Ribonucleic acid
RNase	Ribonuclease
RT-PCR	Reverse transcriptase-polymerase chain reaction
RT-qPCR	Reverse transcriptase-quantitative polymerase chain reaction
SSC	Sodium citrate solution
TB	Tris buffer
TBS	Tris buffer saline
YAd	Young adult animals
